# Enzymatic Hydrolysis of Broken Rice Protein: Antioxidant Activities by Chemical and Cellular Antioxidant Methods

**DOI:** 10.3389/fnut.2021.788078

**Published:** 2021-12-09

**Authors:** Likun Ren, Jing Fan, Yang Yang, Yue Xu, Fenglian Chen, Xin Bian, Tonglin Xing, Linlin Liu, Dehui Yu, Na Zhang

**Affiliations:** Key Laboratory of Food Science and Engineering of Heilongjiang Province, College of Food Engineering, Harbin University of Commerce, Harbin, China

**Keywords:** broken rice, antioxidant activity, Caco-2 cells, ROS, molecular weight

## Abstract

Excessive reactive oxygen species (ROS) is an important cause of aging, and supplementing antioxidants through diet is one of the important ways to delay aging. Some studies have confirmed that rice protease hydrolysate has antioxidant activity, but was rarely been investigated on cells. Thus, commercial enzymes, alkaline enzyme, neutral enzyme, pepsin, chymotrypsin, and trypsin were selected to hydrolyze broken rice protein (BRP) to obtain the corresponding hydrolysates, which were A-broken rice protein hydrolysate (BRPH), N-BRPH, P-BRPH, C-BRPH, and T-BRPH, respectively. Then the antioxidant properties of BRPHs were evaluated by different chemical and cellular antioxidation. Molecular weight, peptide length distribution, and amino acid sequence were detected to insight into the antioxidant properties. Among BRPHs, the A-BRPH displayed the strongest hydroxyl radical scavenging activity (IC_50_ = 1.159 mg/ml) and metal ion-chelating activities (IC_50_ = 0.391 mg/ml). Furthermore, cellular antioxidation confirmed that A-BRPH significantly increased cell viability and inhibited the intracellular ROS release in both aging cells and cell-aging processes. Sodium dodecyl sulfate-polyacrylamide gel electrophoresis (SDS-PAGE) results revealed that peptides with molecular weight <14.5 KDa were produced by enzymatic hydrolysis. Additionally, A-BRPH rich in low molecular weight (<3 kDa) and short-length peptides with some specific amino acids, such as aromatic and hydrophobic amino acids, contributes to the antioxidant properties. This study provided theoretical to the utilization of broken rice and confirmed that A-BRPH could be used in new anti-aging food and health products for human consumption.

## Introduction

Aging is an unavoidable and complex process (1, 2), which has been defined as a gradual decline process of tissue and organ function by all living beings over time. Many theories, such as the mitochondrial damage, telomere shortening, and the increased free radical content, have been proposed to sustain the aging (3–5), in which the free radical theory has been supported by a large number of researchers. The free radical theory of aging was put forward by Denham Harman in the 50s of the twentieth century that free radicals produced during the metabolism of the body result in aging (6). Reactive oxygen species (ROS) is an unavoidable free-radical product in aerobic organisms during respiration (7), such as hydroxyl, superoxide anion radicals, hydrogen peroxide, and so on, playing a significant role in cell signal transduction and homeostasis. Excessive ROS can damage biomacromolecule (DNA, lipids, and protein), resulting in oxidative stress caused by aging, related diseases, such as cardiovascular disease, Parkinson's diseases, would happen (8, 9). The excess free radicals can be scavenged by antioxidants, and those from dietary were effective strategies to attenuate the ROS deleterious effects (10). Hence, significant interest has been drawn to generate food-derived natural antioxidants.

Rice (*Oryza sativa*), labeled as a valuable protein because of its hypoallergenic and high nutritional characteristics, is used as a major grain source of food especially in Asian countries with a population half about the world (11). About 14% of the rice will be broken during rice dehulling and polishing, and the polished rice was usually sold as a premium product, however, its by-products have been underexploited, such as broken rice. Moreover, the huge demand for rice results in incommensurate amount of rice by-produce (12). The broken rice, which was plentiful and readily available, can be used to produce valuable food ingredients as one of the new directions in food research (13). Previous studies have shown that protein hydrolysates from milk, egg, soybean, marine fish proteins, etc., exhibit antioxidant activities (14, 15). Some researchers have discussed the antioxidant capacity of the broken rice protein hydrolysate (BRPH), but little information is available on the effect of cell aging or aging cells (16).

Human intestine as a primary digestive system has digestive enzymes, and extreme pH environments could impact the functionalities of the food. Thus, considerable attention has been spawned to assess the antioxidant effect of natural compounds on the intestinal epithelium. Human colon cancer cells (Caco-2 cells) injured by hydrogen peroxide (H_2_O_2_) have been regarded as a reliable model to explore the physiological response of intestinal epithelium to food-derived natural antioxidants (17).

Hence, BRPH was prepared by five commercial proteases, and the antioxidant properties were determined by different methods, namely, 1,1-diphenyl-2-picrylhydrazyl (DPPH), hydroxyl scavenging activities, and chelating activity of Fe^2+^. The cytoprotective and antioxidant effects against H_2_O_2_-stressed Caco-2 cells were evaluated by inhibiting intracellular ROS. In addition, we also measured the molecular weight and peptide length distribution of the hydrolysates with sodium dodecyl sulfate-polyacrylamide gel electrophoresis (SDS-PAGE) and liquid chromatography-mass spectrometry (LC-MS) techniques to separate and purify the anti-aging peptide.

## Materials and Methods

### Materials

Broken rice was supported by Jinhe Group Co., Ltd. (Herbin, China). Alkaline protease from *Bacillus licheniformis*, Neutral proteinase, Trypsin, α-amylase, and Glucoamylase were purchased from Yuanye Bio-Technology Co., Ltd (Shanghai, China). Pepsin and Chymotrypsin were obtained from SUMMUS biological technology co., Ltd. (Harbin, China). DPPH, 2,2′-azinobis (3-ethylbenzothiazoline-6-sulfonic acid) diammonium salt (ABTS), and ferrozine were donated by Sigma Chemical Co. (St. Louis, MO, USA). The Caco-2 cells, derived from human colon adenocarcinoma, were purchased from the American Type Culture Collection (ATCC). Dulbecco′s modified Eagle medium (DMEM, high glucose), fetal bovine serum (FBS), penicillin-streptomycin, and 2′, 7′-dichlorofluorescin diacetate (DCFH-DA) were obtained from Solarbio Science & Technology Co., Ltd. (Beijing, China). A Cell Counting Kit-8 (CCK-8) was obtained from Beyotime Biotechnology (Shanghai, China). All the other chemicals used in this study were analytical grade.

### Preparation of Defatted Broken Rice Powder

The sample was crushed, sifted, and defatted according to the method with some modifications (18). The sample was dispersed in n-hexane at a ratio of 1:4 (*w*/*v*) and stirred for 4 h at 25°C. Subsequently, the residue was collected by vacuum filtration and degreased twice. The defatted broken rice was left overnight and stored at 4°C for further use.

### Determination of Optimum Protein Extraction Method

The optimum protein extraction method was determined by evaluating the purity and the content of the protein product using the Kjeldahl method, which was calculated by the following equation:


$$\begin{array}{l} \text{Protein yield (\%)}=\frac{\text{Protein content in supernatant}}{\text{protein content in Broken rice}}\times 100\\ \text{Rice protein purity (\%)}=\frac{\text{Grams of protein in products}}{\text{Grams of product}}\times 100 \end{array}$$

#### Alkaline Extraction Method

A total 50.0 g Broken rice flour was soaked in 500 ml 0.05 mol/L NaOH according to the description of Hou et al. (19). In brief, the mixture was continuously stirred for 1 h at 25°C and then centrifuged at 4,000 r/min for 15 min. The supernatant was recovered and adjusted to the pH to isoelectric (4.6) using 1 mol/L HCl, rested for 12 h, and centrifuged again. The precipitate was neutralized and then freeze-dried under vacuum at −60°C for 48 h. Finally, the obtained powder was referred to as BRP.

#### Protein Extracted by the Enzyme-Alkali Method

An enzyme impurity removal and alkaline extraction method were performed on the reported by Jianhua et al. (20) with slight modifications. First, 50.0 g of defatted broken rice powder was dissolved in 500 ml with distilled water and stirred for 1 h at 90°C (contain α-amylase, pH = 6.2). Afterward, the final pH was adjusted to 4.5 and temperature to 55°C. Glucoamylase was added to the sample to conduct hydrolysis for 2 h. The mixture was heated in boiling water for 10 min to inactivate the enzyme and then centrifuged at 4,000 r/min for 15 min. The residue was treated using alkali dissolving acid sedimentation according to the method described in section Alkaline extraction method. Finally, the protein prepared by the amylase-alkali method was obtained.

### Preparation of BRPHs

The BRP was hydrolyzed with five different commercial enzymes—Alkaline, Neutral, Pepsin, Chymotrypsin, Trypsin—at their respective optimum conditions as the list in Table 1. Total 1.0 g BRP powder was dispersed in 20 ml distilled water to form a solution and adjusted to appropriate pH and temperature for hydrolysis (displayed in Table 1). Then the hydrolysis was initiated by adding enzymes (10,000 U/g) lasting for 3 h with constant pH and temperature. After incubation, the enzymes in the mixture were stopped by heating at 95°C for 10 min and centrifuged at 4,000 r/min for 15 min at 4°C. The supernatants were freeze-dried and stored at −4°C (21).

**Table 1:** Optimal hydrolysis conditions.

	**Temperature (**°**C)**	**pH**
Alkaline	40	10.5
Neutral	45	7.4
Pepsin	37	1.5
Chymotrypsin	25	8.0
Trypsin	37	8.1

### Determination of Degree of Hydrolysis (DH)

The DH was determined using the ortho-phthalaldehyde (OPA) method (22). The serine was dissolved in water to final concentrations of 0, 0.02, 0.04, 0.06, 0.08, and 0.1 mmol/L, then OPA was added for 2 min and detected by Ultraviolet spectrophotometer (400 nm) for drawing the serine standard curve. The OPA regent was mixed with 160 mg OPA (dissolved in 4 ml ethanol), 200 mg SDS, 176 mg DTT, 7.62 g sodium, and distilled water (200 ml).

One milliliter BRPH was diluted 100 times with deionized water. Subsequently, 400 μl diluted BRPHs were mixed with 3 ml OPA, after 2 min, the absorbance of the mixture was determined at 400 nm. The results were expressed as:


$$\begin{array}{l} DH\left(\%\right)=\frac{\text{h}}{{\text{h}}_{\text{tot}}} \end{array}$$

Where h was calculated by the OD of serine standard, htot was 7.44 mequiv/g of rice.

### Antioxidant Capacity Assays by Chemical Methods

The radical-scavenging capacity, such as DPPH, hydroxyl, and chelating activity of Fe^2+^ with IC_50_, was obtained to assess the antioxidant ability by chemical methods.

#### DPPH Radical Scavenging Activity

The DPPH radical-scavenging activity of BRPHs was determined by the method described by López-Pedrouso et al. (23) with some modifications. Briefly, 4 ml of 0.1 mmol/L DPPH solution (in 95% ethanol) was thoroughly mixed with 1 ml BRPHs at different concentrations (0.1, 0.5, 1, 2, 2.5, 5, 10, and 25 mg/ml). The mixture with vigorous shaking and incubated at 25°C in dark for 0.5 h, followed by using a spectrophotometer to detect the mixture absorbance at 517 nm (T6, Beijing Puxi General Instrument Co., Beijing, China). Positive control was performed using Glutathione (GSH). The DPPH radical scavenging activity was calculated as follows:


$$\begin{array}{l} \text{DPPH scavenging capacity (\%)}=\frac{A_{blank}-\left(A_{sample}-A_{control}\right)}{A_{Blank}} \end{array}$$

Where A_blank_ is the absorbance of the ethanol, DPPH, and distilled water; A_sample_ is the absorbance of the BRPHs; A_control_ is the absorbance of the sample without DPPH.

#### Ferrous ion Chelating Activity

The metal chelating activity of BRPHs was measured by the modified method of Pino et al. (24). Total 1.96 ml sample solution of different concentrations (0.1–25 mg/ml) was mixed with 0.28 ml of 0.2 mmol/L Fecl_2_. After a water bath for 3 h at 37°C, 0.56 ml of 5 mmol/L ferrozine was added, and then it was incubated for 10 min. Finally, the absorbance of the mixture was measured at 562 nm. The results were expressed as:

$$\begin{array}{l} \text{Ferrous ion chelating activity (\%)}=1-\frac{A_{sample}-A_{control}}{A_{blank}} \end{array}$$

Where A_sample_ is the absorbance of BRPH; the A_control_ is the absorbance of a sample without ferrozine; and the A_blank_ is water instead of the sample.

#### Hydroxyl Radical Scavenging Activity

The hydroxyl radical scavenging activity of BRPH was determined using the method of Mu et al. (25) with some modifications. 50 μl of FeSO_4_ (1.8 mmol/L), 50 μl of 1.8 mmol/L H_2_O_2_ were added to 50 μl different concentrations (0.1–25 mg/ml) of BRPH in turn, then vigorous shaking. Afterward, the mixture was kept at room temperature for 10 min. Then added the salicylic acid to the mixture and measured the absorbance at 510 nm after standing for 30 min. GSH was used as a positive control. The abilities of Hydroxyl radical scavenging were calculated as the following:

$$\begin{array}{l} \text{Hydroxyl radical scavenging activity (\%)}=1-\frac{A_{sample-}A_{control}}{A_{blank}} \end{array}$$

Where A_sample_ is an absorbance in the presence of the sample, A_control_ is the absorbance of the control solution without salicylic acid, and A_blank_ is the absorbance of the solution in the absence of the sample.

### Cellular Antioxidant Activity Assay

#### Cell Culture

Caco-2 cells were cultured in DMEM supplemented with 1% penicillin-streptomycin and 10% fetal bovine serum at 37°C in the 5% CO_2_ incubator (Haier, Qingdao, China). The fresh medium was changed every other day until cells at the concentration of 80%−90% were subcultured by trypsin-EDTA treatment and transferred to different cell culture flasks (26). Cells exposed to a 200 mmol/L H_2_O_2_ solution in DMEM for 1 h were established as a model. The control cells were treated with medium only.

#### CCK-8 Assay

The cell viability was measured by the CCK-8 kit (27). Briefly, Caco-2 cells were seeded in a 96-well plate at a density of 2 × 10^3^ cells/well. After 24 h, the medium was aspirated out, and then the cells were washed with PBS.

To test the cell cytotoxicity effect, the cells were treated with the medium containing BRPHs (0.01–15 mg/ml) for 24 h, then the cells were rinsed three times with PBS. To test the effect of BRPHs on alleviating cell aging, the cells were treated with various concentrations (0.05, 0.25, and 1.00 mg/ml) of BRPH medium for 24 h, then the Caco-2 cells were exposed to 200 mmoL/L H_2_O_2_ for 1 h. To test the effect of BRPHs on senescent cells, the cells were first exposed to 200 mmoL/L H_2_O_2_ for 1 h, then the BRPH medium (0.05, 0.25, and 1.00 mg/ml) was used on the cells for 24 h. Afterward, the medium was gently removed; the cells were then washed three times with PBS and a fresh DMEM medium containing 10% CCK-8 was added and incubated for 1 h to measure the absorbance. The absorbance was measured using a microplate reader (Molecular Device SpectraMax i3x, Sunnyvale, CA, USA) at 450 nm. The results were determined as follows:

$$\begin{array}{l} \text{Cell viability (\%)}=\frac{A_{sample}-A_{blank}}{A_{control}-A_{blank}} \end{array}$$

Where A _blank_ was the absorbance of the mixture without cells, and A _control_ was the absorbance of the mixture without sample.

#### Intracellular Accumulation of ROS

The intracellular ROS production of the cells treated with BRPH was measured with an oxidation sensitive dye 2′,7′-dichlorofluorescein diacetate (DCFH-DA) (28). Cells (5 × 10^3^ cell/well) growing in 96-well plates were loaded with 100 μl DMEM medium per well and allowed to attach for 24 h at 37°C in a carbon dioxide incubator. The Caco-2 cells were pretreated with 0.05, 0.25, and 1.00 mg/ml BRPHs for 24 h, and then 200 μmol/L H_2_O_2_ was added to induce acute oxidative stress. The DMEM was replaced with 10 mmol/L DCFH-DA and cultured in dark for 20 min. The cells were washed with DMEM (contains no FBS) until the extracellular probe was removed. The cells were resuspended with the serum-free medium, and the fluorescence intensity was measured at 485 and 535 nm of the excitation wavelength (Ex) and the emission wave-length (Em), respectively.

The cytoprotective effect of protease hydrolysate restores oxidative stress induced by H_2_O_2_ was also measured. For this purpose, the cells were treated with 200 μmol/L H_2_O_2_ for 1 h before the BRPHs were added.

#### Distribution of Molecular Weight and Peptide Length

Sodium dodecyl sulfate-polyacrylamide gel electrophoresis was performed at 12.5% (V/V) hand-cast polyacrylamide gels. To evaluate the protein molecular weights, 8 μl of the Marker, 10 μl 10 mg/ml BRPHs were loaded and were run at 100 V for 120 min at 25°C. The gels were stained with Coomassie Blue Fast Staining Solution and de-stained in water. The SDS-PAGE image was analyzed by an image analysis system.

Molecular weight and peptide length distributions of A-BRPH were determined using the Triple TOF 5,600 + LC/MS system (AB Sciex, Redwood City, CA, USA). Briefly, the freeze-dried A-BRPH sample was diluted to 1 μg/μl with the machine buffer. The peptide solution was added to the C18 capture column (3, 350 μm × 0.5 mm, AB Sciex, USA), and the C18 analytical column (3, 75 × 150 μm) was applied with a 60 min time gradient and a flow rate of 300 ml/min. mm (Welch Materials, Inc.) for gradient elution. The mobile phase consisted of buffer A (2% acetonitrile/0.1% formic acid/98% H_2_O) and buffer B (98% acetonitrile/0.1% formic acid/2% H_2_O). For Information Dependent Acquisition (IDA), the MS spectrum is scanned with an ion accumulation time of 250 ms, and the MS spectrum of 30 precursor ions is acquired with an ion accumulation time of 50 ms, collect MSI spectrum in the range of 350–1,200 m/z, and collect MS2 spectrum in the range of 100–1,500 m/z. Set the precursor ion dynamic exclusion time to 15 s. Afterward, the original MS/MS files from the mass spectrometer were submitted to Protein Pilot (https://sciex.com. cn/products/software/protein pilot-software, version 4.5, SCIEX, Redwood City, California, USA) for data analysis. The following parameters were considered: the cysteine is modified with iodoacetamide; the biological modification is selected as the ID focus. For the identified protein results, select certain filtering criteria, and peptides with an unused score >1.3 (a credibility of more than 95%) are considered credible peptides, and proteins containing at least one unique peptide are retained.

### Statistical Analysis

All data were presented as mean ± SD at least three independent experiments. The statistical analysis was performed by SPSS 26 (SPSS Inc., Chicago, IL, USA), and the figure was drawn using GraphPad Prism 9 (GraphPad Software, San Diego, CA, USA) for windows.

## Results and Discussion

### Determination of the Optimal Protein Extraction Method

The alkaline extraction method and amylase-alkali method were used to extract BRP and screened the best extraction method with the purity and the extraction percent. As shown in Table 2, the protein yield and purity of BRP extracted by alkaline extraction method were 80.34 ± 0.02% and 90.50 ± 0.01, which were better than the corresponding results of the amylase-alkali extraction method (54.57 ± 0.06, 70.05 ± 0.09). The extraction effect of the alkaline extraction method was 1.65-fold compared with that of the enzyme-alkali method, related to the high gluten content in the protein composition of broken rice. The majority of rice protein was in the gluten fraction (80%), which shows higher solubility in alkali media (29). Gluten of rice exists in cells as a protein bodies-II (PB-II), crosslinking through intermolecular secondary bonds, and are wrapped by starch molecules. Alkali treatment of protein reduces interactions between protein, followed by loosening the structure of protein and starch molecules, promoting the extraction of protein (30). Furthermore, rice protein existed inside the starch, which means there was a need to remove starch molecular to release the protein. Therefore, amylase was used to pretreatment broken rice before alkaline extraction and acid precipitation to improve the extraction rate. In theory, the protein extraction rate and purity obtained by the amylase-alkali method are better than that by a single alkali solution and acid precipitation method, whereas the extraction yield and purity were declined by 32.07 and 22.59% in this study, respectively. The difference in the result between this study and the expected could be due to the effect of enzymatic hydrolysis temperature on protein structure. High temperature could denature the proteins, contribute to the formation of disulfide cross-linking, enhance the interaction between protein and starch result in the solubility increase. Therefore, in this study protein could be removed with the starch suspension after centrifugation, resulting in the reduction of the purity and extraction rate of the protein.

**Table 2:** Extraction ratio and purity of protein from broken rice.

	**Alkaline extraction and acid precipitation**	**Amylase-alkali**
Extraction yield (%)	80.34 ± 0.02	54.57 ± 0.06
Purity (%)	90.50 ± 0.01	70.05 ± 0.09

### Preparation of Protease Hydrolysate and DH

**Figure 1:**
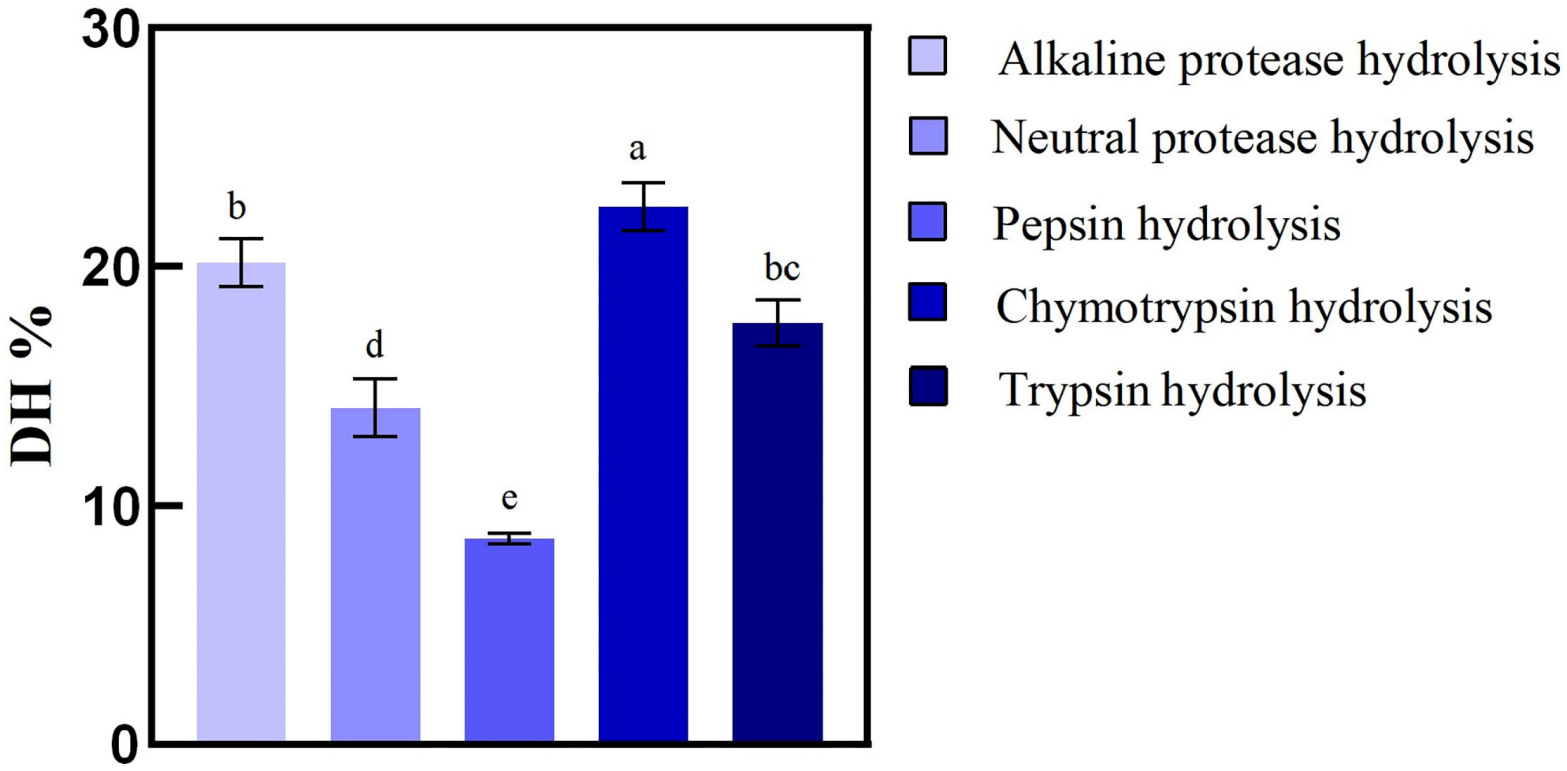
Hydrolysis degree of broken rice by different proteases. Values are shown as mean ± SD (*n* = 3). Different letters (a–d) over a bar indicate significant differences (*p* < 0.05) in four proteases.

The types of hydrolyzing proteins proteases were an important parameter to evaluate the antioxidant capacity of enzymatic hydrolysate. Previous studies have shown that antioxidant characteristics and radical-scavenging activities of the hydrolysates could be influenced by the DH value (31). Five kinds of commercial proteases selected to evaluate BRP were used to produce specific hydrolysates/peptides with strong antioxidant activity that could alleviate the ROS release as an antioxidant. The DHs of hydrolysate of BRP were 20.17, 18.70, 8.62, 22.50, and 15.79% for Alkaline, Neutral, Pepsin, Chymotrypsin, and Trypsin, respectively. As shown in Figure 1, Chymotrypsin was found to be the most efficient, followed by Alkaline, Neutral, Trypsin, and Pepsin. The peptide bonds cleaved more efficiently likely related to the pH of hydrolysis. An alkaline environment could contribute to rice protein dissolution, promoting the enzyme to combine with the substrate, improving the degree of peptide bond breaking, and increasing the DH (32). Among the five proteases, the alkaline enzyme exhibits remarkably higher hydrolytic efficiency than others, except chymotrypsin. The high DH value of alkaline protease was related to that most rice proteins were glutelin dissolved in alkaline solution with the optimum pH reaction range. Our findings were also comparable to those of Zhou et al. (33), that alkaline protease has higher enzymatic hydrolysis efficiency when hydrolyzing rice protein. On the contrary, the condition of pepsin hydrolysis pH was 1.5, with which most proteins were not dissolved, so DH values were low. Moreover, the cleavage sites of alkaline protease (Ala-, Leu-, Val-, Tyr-, and Phe-) had broader specificity than the other four hydrolases were also a crucial reason. Generally, peptide content, peptide chain length, and structure were affected by the DH and influenced the functional activity. Therefore, the chemical antioxidant activity of the BRPHs could be evaluated.

### Antioxidant Activities Assay

**Figure 2:**
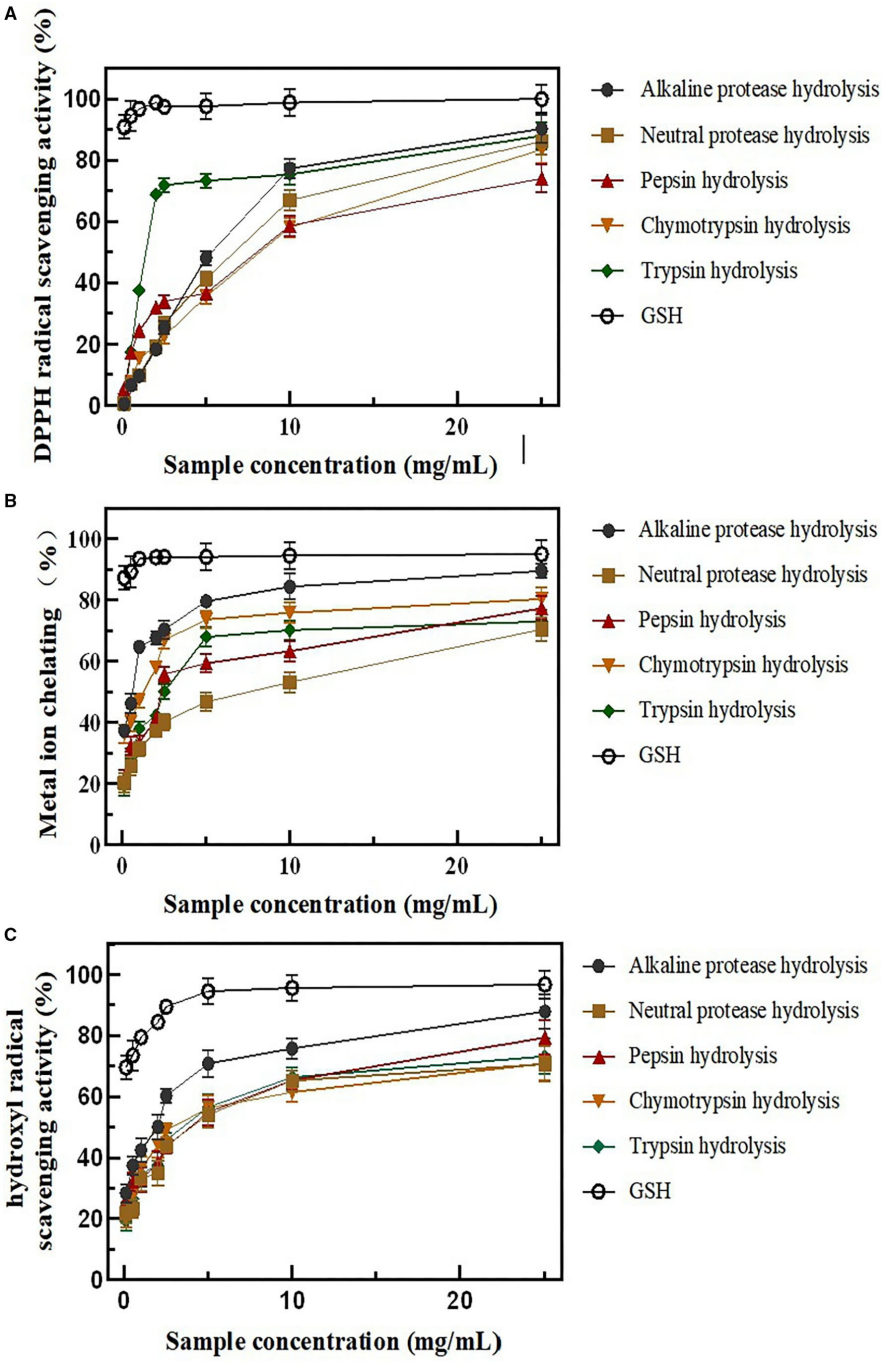
BRPHs antioxidant capacity *in vitro* **(A)** DPPH radical scavenging activity; **(B)** metal ion-chelating activity; **(C)** hydroxyl radical scavenging activity; Data are expressed as mean ± SD, *n* = 3. BRPHs, BRPH, broken rice protein hydrolysate; DPPH, 1,1-diphenyl-2-picrylhydrazyl.

The antioxidant properties with various concentrations (0.1, 0.5, 1.0, 2.0, 2.5, 5.0, 10.0, and 25.0 mg/ml) of all five rice protein hydrolysates were evaluated by chemical assays, such as DPPH scavenging activity, hydroxyl scavenging activity, and Fe^2+^chelating activity. The control was performed using GSH (Figure 2).

DPPH is a stable free radical, which reacts with compounds that can donate a hydrogen atom and has been widely used to test the antioxidant ability of antioxidants (34). As Figure 2A describes, all of the BRPHs have a DPPH radical scavenging activity and show concentration related. However, the DPPH antioxidant capacity of GSH was significantly higher than BRPHs with all concentrations. Moreover, the half-inhibition concentrations (IC_50_) were the BRPHs concentration that could clearance 50% radicals calculated and shown in Table 3. Specifically, the IC_50_ values of A-BRPH, N-BRPH, P-BRPH, C-BRPH, and T-BRPH were 2.997 ± 0.46, 3.961 ± 0.21, 4.589 ± 0.32, 5.148 ± 0.67, and 1.677 ± 0.08 mg/ml, respectively. T-BRPH exhibited notable DPPH radical scavenging ability and followed by A-BRPH, N-BRPH, P-BRPH, and C-BRPH. The differences in radical scavenging activity might be due to the differences in the molecular weight and amino acid composition in hydrolysate (35, 36). The antioxidant samples with low weight and hydrophobic amino acids could easily enter into target organs through hydrophobic interactions with membrane lipid bilayers, where they could exert a significant capacity of scavenging radicals. Moreover, Lapsongphon et al. (37) noticed sulfur-containing amino acids, such as cysteine, can provide hydrogen at a thiol group to improve DPPH radical scavenging ability. Hence, it was speculated that compared with other enzymatic hydrolysates, the peptide in A-BRPH had a small molecular weight and contains hydrophobic and sulfur-containing amino acids.

**Table 3:** The IC 50 value of samples.

		**DPPH radical**	**Metal ion chelating**	**^**.**^OH radical**
Sample	A-BRPH	2.997 ± 0.46	0.391 ± 0.01	1.159 ± 0.10
	N-BRPH	3.961 ± 0.21	5.810 ± 0.33	4.031 ± 0.33
	P-BRPH	4.589 ± 0.32	2.532 ± 0.20	2.964 ± 0.27
	C-BRPH	5.148 ± 0.67	0.722 ± 0.00	3.225 ± 0.22
	T-BRPH	1.677 ± 0.08	2.371 ± 0.12	3.369 ± 0.16

*All data are presented as the mean ± SD of triplicate results*.

Excess Fe^2+^ could catalyze the production of ROS by Fenton reaction and lead to oxidative damage in biological macromolecules. As seen in Figure 2B, the chelating capacity of BRPHs is found to be dependent on the sample at higher concentrations. The A-BRPH possesses an effective chelating capacity for Fe^2+^. The IC_50_ value of Ferrous ion chelating activity follows as A-BRPH (0.391 ± 0.01 mg/ml) > C-BRPH (0.722 ± 0.00 mg/ml) > T-BRPH (2.371 ± 0.12) > P-BRPH (2.532 ± 0.20) > N-BRPH. These results showed that after enzymatic hydrolysis with alkaline protease, more specific peptide structures with high metal-binding affinity might be formed than other protease hydrolysates, for instance, -SH or/and L-Cys (38).

The hydroxyl radical was an important component of ROS in a biological system, which possess the strongest chemical activity (39). To make the results of antioxidant activity *in vitro* closer to its biological effect, the hydroxyl radical (^.^ OH) scavenging capacities of the BRPHs were measured in this study. Similar to the results for other scavenging capacities, the BRPHs exhibited a dose-dependent increase in the hydroxyl radical scavenging capacities. As shown in Figure 2C, the • OH, scavenging activities of A-BRPH are significantly higher than that in other experimental at various concentrations. The IC_50_ value of the BRPHs was further confirmed that A-BRPH (1.159 ± 0.10) had the highest activity.

In summary, all the enzymatic treatments improved the antioxidant capacities of protein. Moreover, several studies have verified the relationship between peptides chain sequence and length with antioxidant properties. It is widely accepted that short peptides (contain 3–15 amino acids) show higher radical scavenging activity (40). Furthermore, the results of the OH radical and metal ion chelating antioxidant models were similar to each other presumably because they have the same mechanism, to inhibit the Fenton reaction, reducing hydroxyl radical formation and radical chain reactions subsequently. The DPPH assay is based on both electron transfer (SET) and hydrogen atom transfer (HAT) reactions (41). Different reaction mechanisms lead to inconsistency between DPPH and the other two in the three antioxidant evaluation experiments. It is noteworthy that the A-BRPH shows the highest antioxidant activity in hydroxyl radical scavenging assay and ferrous ion-chelating assay, but not in DPPH radical scavenging activity. This phenomenon might be related to the amino acid composition of peptides in BRPHs. The DH is an important index to evaluate the efficiency of enzymatic hydrolysis and affected the antioxidant capacity to an appropriate degree. The higher DH value indicated a more efficient enzymatic hydrolysis, indicating excessive peptide bonds cleavage resulting in amino acids content raised, antioxidant activities reduced. In contrast, the lower DH value indicated that the enzymatic hydrolysis with less efficiency, related to the long peptide chain. Zou et al. (42) pointed out that peptides with long peptide chains showed weaker antioxidant performance, due to the hard entrance to cells as an antioxidant. In comparison, A-BRPH exhibited the best antioxidant properties in this study at the DH of 20.17%, indicating that a large number of radical inhibitory peptides were produced. All these results showed that BRPHs were a potential source of natural antioxidants, further verified by cell antioxidant experiments.

### Effect of BRPHs on Cell-Based Antioxidant Activities

The aging of organisms occurs at all levels of the body, and cell aging is considered to be the basis of body aging. Therefore, the Caco-2 cells were utilized to explore the physiological response of the human intestine epi-thelium to oxidative stress and potential mechanism.

#### Cytotoxicity Assays

A Cell Counting Kit-8 method was used to evaluate the BRPH effects with different concentrations applied on Caco-2 cells.

**Figure 3:**
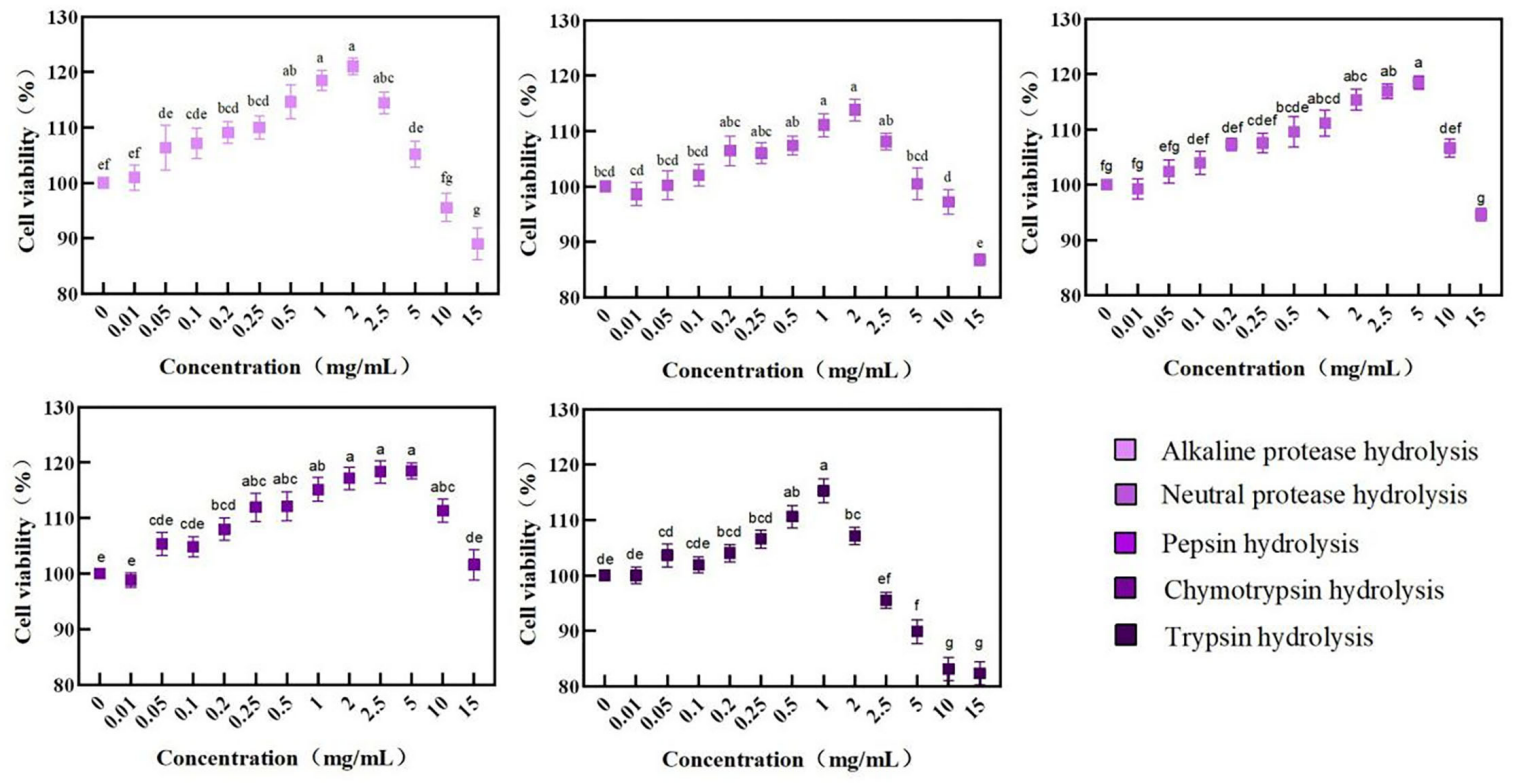
Cell viability of Caco-2 cells treated with BRPHs. Results are expressed as means ± SD (*n* = 4). Different letters (a–g) the picture indicate statistically significant differences (*p* < 0.05).

As shown in Figure 3, the cell viability of all BRPHs is increased firstly and then decreased with the concentration increase. The cell viability decrease was related to the extracellular environment. The high permeability liquid formed with high sample concentration results in excessive water loss and cell death (43). Among them, the cell viability of the Caco-2 was A-BRPH concentration-dependent, but decreased at the concentration exceeding 2 mg/ml. Additionally, when the BRPHs concentration was 2 mg/ml (N-BRPH), 5 (P-BRPH), 5 (C-BRPH), and 1 mg/ml (T-BRPH), the cell viability was higher, respectively. Cell viability ≥100% meant BRPHs had no cytotoxicity to Caco-2 cells. In order to compare the effects of BRPHs acted on cells, BRPH concentrations of 0.05, 0.25, and 1.00 mg/ml were selected for the next experiment.

#### BRPHs Delayed the Aging of Caco-2 Cells

**Figure 4:**
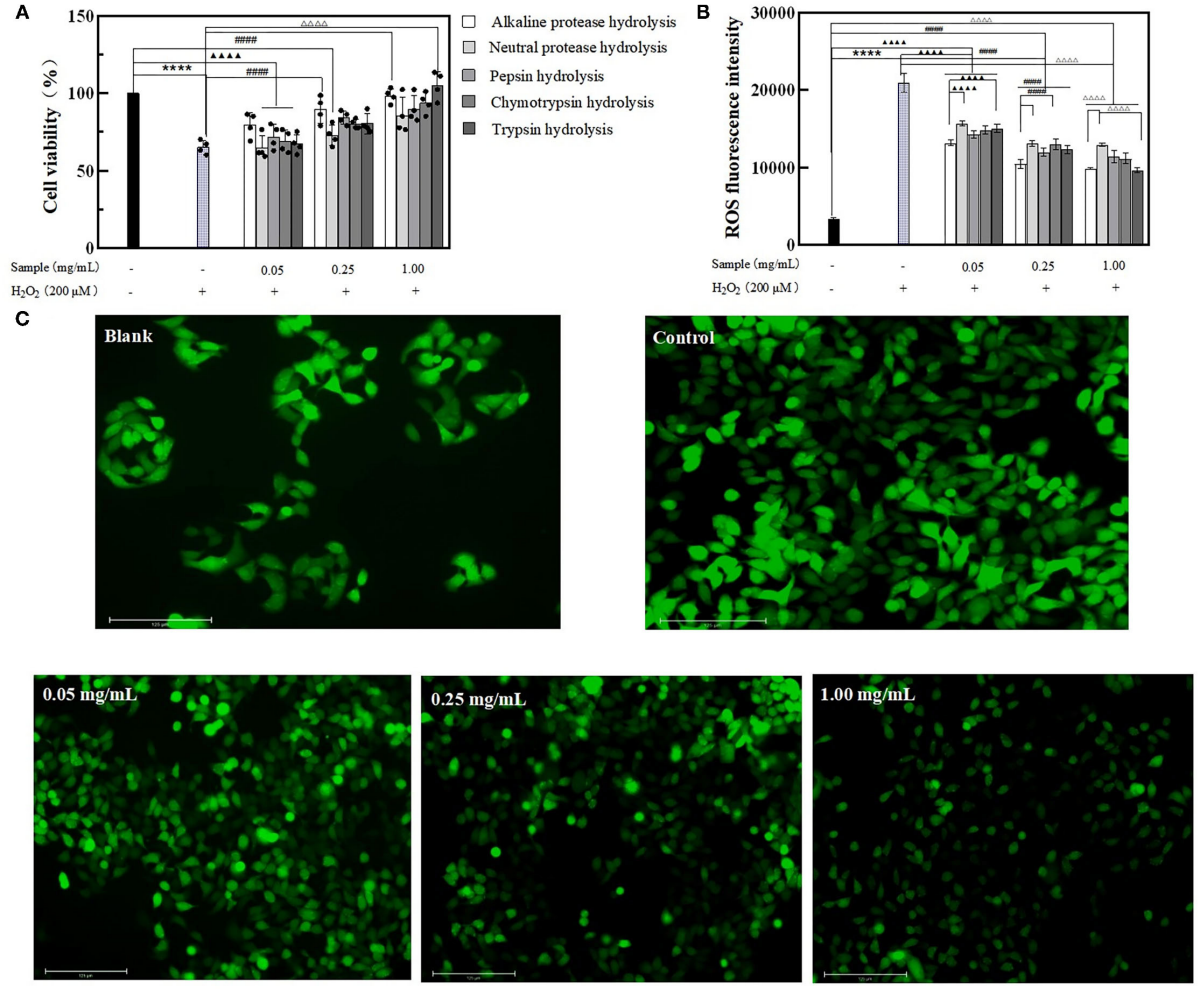
The effect of BRPHs on alleviating cell aging. **(A)** cell viability; **(B)** ROS fluorescence intensity; **(C)** ROS fluorescence image of Caco-2 cells treated with 0.05, 0.25, and 1.00 mg/ml of A-BRPH. Cells were pretreated with the sample at 0.05, 0.25, 1.00 mg/ml for 24 h before exposure to 200 μm H_2_O_2_ for 1 h. Data were shown as mean ± SEM, *n* = 4. ANOVA: ****, *p* < 0.0001 compared with control; ▴▴▴▴, *p* < 0.0001 compared with Caco-2 cells treated with 0.05 mg/ml sample for 24 h; ####, *p* < 0.0001 compared with Caco-2 cells treated with 0.25 mg/ml sample for 24 h; ΔΔΔΔ, *p* < 0.0001 compared with Caco-2 cells treated with 0.25 mg/ml; sample for 24 h. BRPH, broken rice protein hydrolysate.

Pretreated the Caco-2 cells with BRPHs at low (0.05 mg/ml), medium (0.25 mg/ml), high (1.00 mg/ml) concentrations, then the cells were exposed to H_2_O_2_ used to induce oxidative stress, forming the normal aging process of cells (8). To clarify the effect of hydrolysate on cell aging, the cell viability and the accumulation of ROS within the cells is investigated displayed in Figure 4A. Caco-2 cells treated with H_2_O_2_ only (Model group) showed low cell viability compared with the other groups, whereas cells pretreated with BRPHs showed protective effects. An increase in hydrolysate concentration improving the cell viability was consistent with the reported by Zhang et al. (44). The higher amount of antioxidant peptides in the high BRPH could be a reason for this phenomenon. Among the five hydrolysates, A-BRPH exhibited optimum cytoprotection, and A-BRPH at 0.05, 0.25, 1.00 mg/ml showed significantly increased effects on cell viability (from 65.26 to 79.63%, 89.50, and 98.48%), respectively. In the low dosage group, the highest cell viability values corresponded to samples A-BRPH (79.63%), followed by P-BRPH (71.91%), C-BRPH (69.13%), T-BRPH (67.28%), and N-BRPH (64.81%). The cell viability in descending order was A-BRPH (89.50%) > P-BRPH (84.26%) > T-BRPH (80.56%) > C-BRPH (80.25%) > N-BRPH (72.83%) at a sample concentration of 0.25 mg/ml. As for the high dosage group, the highest cell viability was obtained when the protein was hydrolyzed by trypsin. Moreover, comparing the value of cell viability, pre-incubated with A-BRPH and T-BRPH did not show statistically significant differences.

The effect of hydrolysate on intracellular ROS content was then investigated using a DCFH-DA probe assay. DCFH-DA is a membrane-permeable probe without fluorescence that can be hydrolyzed by esterase to produce DCFH, which cannot penetrate the cell membrane and was loaded into the cell. The intracellular DCFH was then oxidized by ROS to form the fluorescent DCF. Hence, the level of ROS in cells can be known by detecting the fluorescence of DCF. ROS content was positively correlated to fluorescence intensity (45). As seen in Figure 4B, ROS fluorescence intensity has increased significantly when exposure to H_2_O_2_, indicating that H_2_O_2_ had a strong effect on ROS generation. As expected, pretreatment cells with BRPHs remarkably suppressed ROS formation with dose-dependent. This observation is consistent with the report by Zhang (46), who found that cells were treated with alkaline protease-hydrolyzed soybean protein hydrolysate significantly suppressed ROS formation. In this study, compared with the control the ROS levels in treated groups were reduced by 37.18% (A-BRPH), 25.14% (N-BRPH), 31.97% (P-BRPH), 29.29% (C-BRPH), and 28.16% (T-BRPH) respectively at a BRPHs concentration of 0.05 mg/ml. The fluorescence intensity was observed with a remarkable decrease in the high-concentration group from 20,970 to 9,662 in the group that treated with trypsin, followed by alkaline protease (20,970–9,858), Chymotrypsin protease (20,970–11,153), Pepsin protease (20,970–11,416), and neutral protease (20,970–12,973). Overall, A-BRPH could efficiently inhibit the accumulation of ROS in cells. Other studies have investigated those peptides with low MW and short-chain had stronger antioxidant stress activity in the cellular environment (38). The result agreed with the finding of the chemistry oxidant in this study, suggesting that A-BRPH contains a large number of short-peptides with antioxidant activity. Furthermore, fluorescence images were obtained to insight into the effect of A-BRPH on intracellular ROS accumulation (Figure 4C), where higher fluorescent signals implied higher intracellular ROS content. The brightest picture was observed for the cells treated with H_2_O_2_ only, whereas the 1.00 mg/ml A-BRPH-treated cells tended to have a milder brightness than the cells treated with low A-BRPH. These results provide evidence that A-BRPH can effectively protect Caco-2 cells against ROS-mediated oxidative damage, showing concentration-dependent. In addition, the anti-aging effect of hydrolysate was attributed to the reduced ROS content.

#### BRPHs Relieve H_2_O_2_-Induced Damage in Caco-2 Cells

In order to explore the effect of hydrolysate on senescent cells, the aging model of Caco-2 cells was constructed by exposing them to 200 μmol/L H_2_O_2_ for 1 h, and then treated with the BRPHs for 24 h.

**Figure 5:**
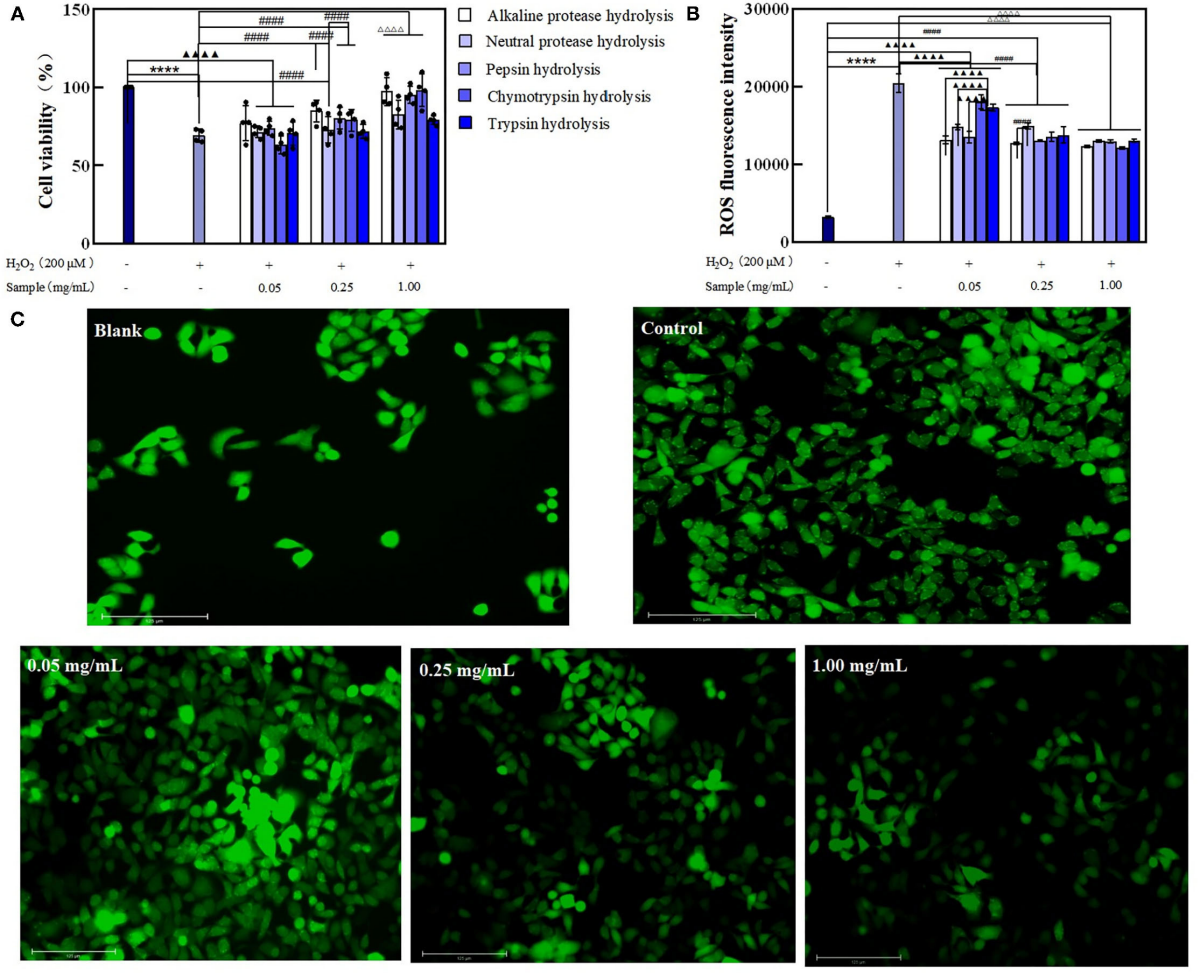
The effect of BRPHs on senescent cells induced by H_2_O_2_. **(A)** cell viability; **(B)** ROS fluorescence intensity; **(C)** ROS fluorescence image of Caco-2 cells treated with 0.05, 0.25, and 1.00 mg/ml of A-BRPH. Cells were pretreated with the sample at 0.05, 0.25, 1.00 mg/ml for 24 h before exposure to 200 μm H_2_O_2_ for 1 h. Data were shown as mean ± SEM, n = 4. ANOVA: ****, *p* < 0.0001 compared with control; ▴▴▴▴, *p* < 0.0001 compared with Caco-2 cells treated with 0.05 mg/ml sample for 24 h; ####, *p* < 0.0001 compared with Caco-2 cells treated with 0.25 mg/ml sample for 24 h; ΔΔΔΔ, *p* < 0.0001 compared with Caco-2 cells treated with 0.25 mg/ml sample for 24. BRPH, broken rice protein hydrolysate.

After the H_2_O_2_-induce cell oxidative stress, cell viability was decreased compared with the control. The cell viability in treated groups was observed superior to that in the model, indicating BRPHs could relieve H_2_O_2_ induced cell viability. Specifically, the cell viability presented the maximum when treated with 0.05 mg/ml A-BRPH, 0.25 mg/ml A-BRPH, and 1.00 mg/ml C-BRPH. When the model cells were treated with 0.05 mol/L samples, A-BRPH, N-BRPH, and P-BRPH treated cell viabilities were increased by 11.66, 3.47, and 6.92%, respectively, compared to the model group (Figure 5A). It is noteworthy that there was no significant change in cell viability after C-BRPH and T-BRPH treatment. This phenomenon could be explained by the fact that H_2_O_2_ induced the cells to produce oxidative stress, destroyed the normal metabolism of the body, and reduced cell viability. Low concentration BRPHs contain fewer antioxidant components could not repair the cell damage. Therefore, we speculated that there were few antioxidant peptides in 0.05 mg/ml C-BRPH and T-BRPH, and could not recover the damage to cells induced by H_2_O_2_. In the medium dosage of 0.25 mg/ml, the highest values corresponded to the sample of A-BRPH, followed by P-BRPH, C-BRPH, N-BRPH, and T-BRPH. Similarly, the ability to mitigate H_2_O_2_ damage of A-BRPH was the highest at 1.00 mg/ml experiment group.

The intracellular generation of ROS was used to evaluate the damage caused by oxidative stress and the ability of the hydrolysates to repair oxidative damage. The results obtained are shown in Figure 5B. The induction of oxidative stress resulted in a significant increase (up to 20,486) in ROS fluorescence intensity vs. the blank (3,240). Moreover, ROS accumulation at the test concentrations of all the BRPHs treated groups was found significantly reduced. This suggested that BRPHs could relieve the damage of cells injured by H_2_O_2_. Compared with the fluorescence intensity of ROS after treating cells with different BRPHs, it was found that A-BRPH could significantly reduce the accumulation of ROS in cells. The Fluorescence images captured by Thermo AUTO 2 was shown in Figure 5C, it can be seen that ROS production could be inhibited by A-BRPH with concentration-dependent. Furthermore, these results were consistent with the finding of cell viability in this study that A-BRPH had a therapeutic ability on aging cells. These phenomena were in accordance with the finding of Li (47), which shows that antioxidant peptides prepared by protease hydrolysis from protein could promote cell proliferation and reduce the ROS level of H_2_O_2_ -induced in the cell significantly. All of these results (cell-based antioxidant activities) confirmed that BRPH had preventive and alleviating effects on the aging cell, in which the A-BRPH effect was significant. This conclusion was in following the determination of antioxidant capacity *in vitro*. The good hydroxyl radical scavenging and ferrous ion-chelating ability of A-BRPH can further explain the results of the cellular antioxidant assay. H_2_O_2_ can be used as a reactant to participate in the Fenton reaction in organisms. When A-BRPH was added to the cell, on the one hand, its ability to complex biologically active transition metals such as Fe^2+^, thus reducing their availability to participate in the Fenton reaction where highly toxic hydroxyl radicals are generated. On the other hand, A-BRPH can reduce the content of hydroxyl radical (Fenton reaction product) (48, 49).

### Distribution of Molecular Weight and Peptide Length

**Figure 6:**
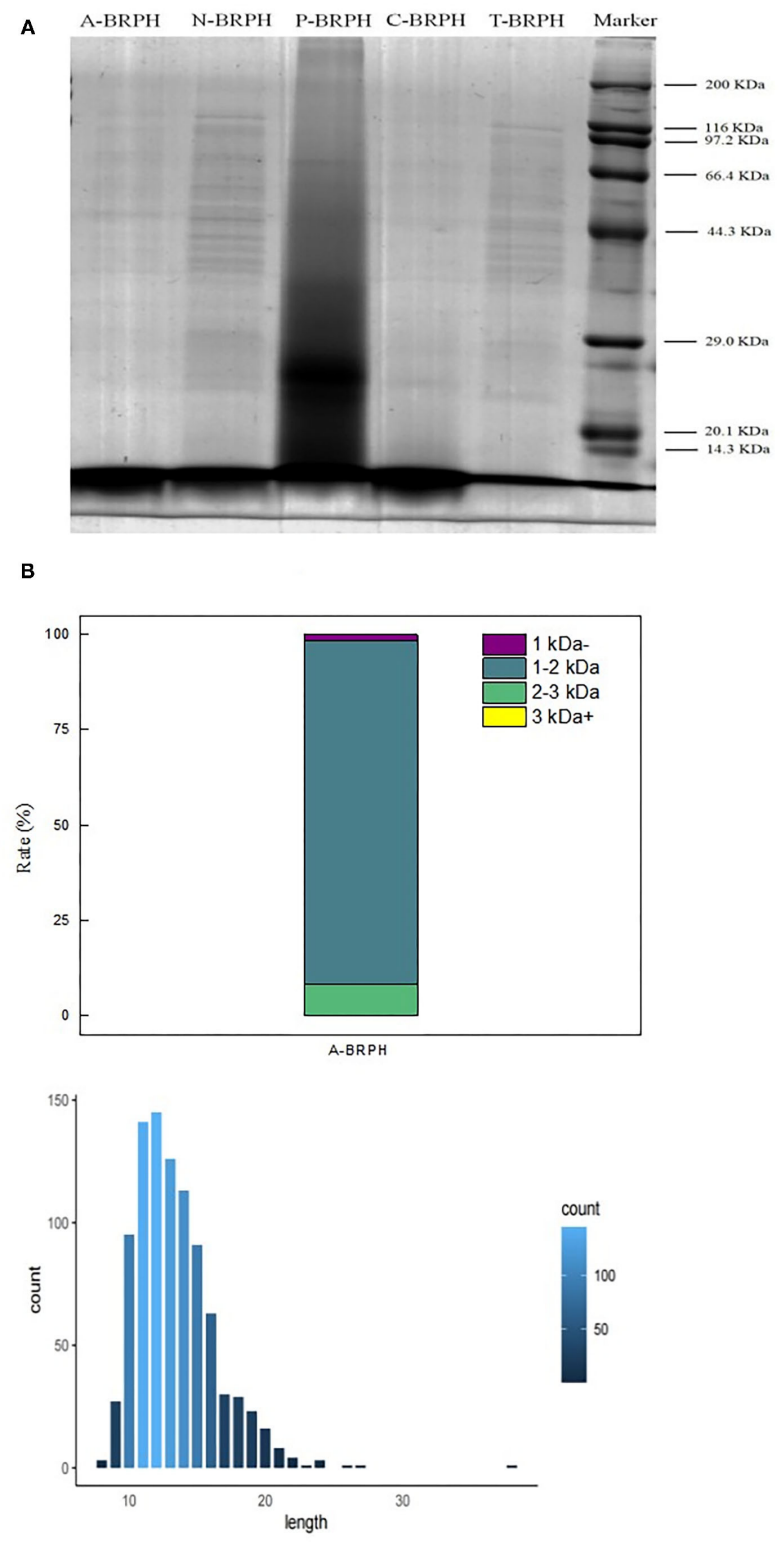
Peptide analysis of A-BRPH. **(A)** SDS-PAGE patterns of BRPHs; **(B)** the molecular weight and peptide length of A-BRPHs. SDS-PAGE, sodium dodecyl sulfate-polyacrylamide gel electrophoresis; BRPH, broken rice protein hydrolysate.

Protein hydrolyzing into peptides could change the length and weight distribution. The SDS-PAGE was used to display the products during the hydrolysates of BRPH to reflect the enzymatic hydrolysis efficiency (Figure 6). Compared with the other experimental groups, there were significant differences in the distribution bands of P-BRPH. The BRP treated with pepsin showed the highest complexity in SDS–PAGE pattern since it mainly showed the presence of proteins in different molecular weight. And there was a large smear at molecular weights (MW) lower than 29 kDa. This result was also comparable to the DH values, which showed that the enzymatic hydrolysis efficiency of pepsin was the lowest. Compared with other Lane, the protein profiles of Lane N-BRPH and T-BRPH were directed between 116 and 44.3 KDa. Moreover, N-BRPH made the band intensity increased with MW lower than 14.3 kDa. Furthermore, Lane C-BRPH and A-BRPH showed significant bands under 14.3 kDa. The length and molecular weight of peptides played a decisive role in antioxidation.

Combined with the results of previous antioxidant experiments, we considered that A-BRPH contained a large number of peptides with high antioxidant activity and the most promising antioxidant activity A-BRPH was assayed for molecular weight and peptide length. As shown in Figure 6B, the A-BRPH contained a large proportion of low-MW peptides and free amino acids are divided into four fractions: <1, 1–2, 2–3, >3 kDa. Additionally, the peptides number of 1–2 kDa accounted for about 90.48% of the total peptides in A-BRPH. In terms of peptides length, there were peptides with 8–40 amino acids and with the length of 11–14 amino acids being predominant, made up ~12.26% of the A-BRPH. Low molecular weight and short peptide chain had better opportunity to react with active radicals in the reaction mixture and provided potential antioxidant effects, where the vital reasons for A-BRPH possessed high antioxidant activity. These results confirmed the reports of Phongthai et al. (50) that described the major antioxidant peptides derived from food protein had hydrolysate consisting of 6–21 amino acids have MW <3kDa.

### Amino Acid Sequence of the Peptide in A-BRPH

The amino acid composition of protein hydrolysates and peptides has been closely correlated with their antioxidant activity (51). It is reported that protein hydrolysates and/or peptides containing amino acid residues such as valine (Val), alanine (Ala), leucine (Leu), phenylalanine (Phe), tryptophan (Trp), methionine (Met), aspartic acid (Asp), proline (Pro), and histidine (His) show higher antioxidant ability (52). In this study, 921 kinds of peptides with the MW range of 854.43–3,263.31 Da in A-BRPH were identified by LC-MS. Among them, 828 kinds of peptides derived from A-BRPH contained one or more of these amino acids, accounting for about 89.9% of the total peptides, which were the main contributor to the antioxidant activity. Specifically, hydrophobic amino acids were associated with strong free radical scavenging activity *via* the direct transfer of electrons. Moreover, it can interact with phospholipid bilayer to make them smoothly enter into target organs and play an antioxidant role **(**50). Hence, A-BRPH exhibits DPPH scavenging activity. About 96 peptides contain Met, which is labeled as an efficient scavenger of reactive oxygen species (e.g., H_2_O_2_, hydroxyl radical). Therefore, it shows activity in the OH radical scavenging experiment in this paper. Moreover, according to Piu et al. (53) and Akinyede et al. (51), acidic or basic amino acids, like Asp, Glu, Arg, and the presence of His residue containing imidazole ring, which was responsible for metal chelation through enhancing electrostatic and ionic interactions with iron. In summary, the presence of these amino acids makes A-BRPH could, to some extent, exert different antioxidant properties in different oxidative systems.

## Conclusions

Results suggested that BRP extracted by alkaline protease showed the highest purity and yield. Moreover, we proved that BRPHs had antioxidant activity. BRPH could pre-protect and repair H_2_O_2_-induced Caco-2 cells from inhibiting the accumulation of ROS in cells. The antioxidant activity of the hydrolysates was significantly improved with an appropriate DH. Alkaline protease hydrolysate (A-BRPH) exhibited excellent antioxidant activity by chemical and cellular assay at 20.17% DH. LC-MS/MS results indicated that A-BRPH content a large number of short-length peptides below 3 kDa with aromatic and hydrophobic amino acids in their sequences, which had the antioxidant ability and could be used as an important source of natural antioxidant for further research. These results might contribute to the utilization of broken rice which was the rice industry by-products and guide further works to purify and identify the effective components of A-BRPH.

## Data Availability Statement

The original contributions presented in the study are included in the article/Supplementary Material, further inquiries can be directed to the corresponding author/s.

## Author Contributions

NZ: supervision and project administration. LKR, YY, and JF: methodology, validation, and writing-original draft and editing. YX and XB: formal analysis. TLX and FLC: data curation and writing-review. LLL and DHY: methodology and validation. All authors contributed to the article and approved the submitted version.

## Funding

We appreciate the financial support from the National Natural Science Foundation of China (32072258), Major Science and technology Program of Heilongjiang (2020ZX08B02), Harbin University of Commerce young innovative talents support program (2019CX06 and 2020CX26), Central financial support for the development of local colleges and Universities. Graduate innovation and scientific research project of Harbin University of Commerce (YJSCX2021-697HSD).

## Conflict of Interest

The authors declare that the research was conducted in the absence of any commercial or financial relationships that could be construed as a potential conflict of interest.

## Publisher's Note

All claims expressed in this article are solely those of the authors and do not necessarily represent those of their affiliated organizations, or those of the publisher, the editors and the reviewers. Any product that may be evaluated in this article, or claim that may be made by its manufacturer, is not guaranteed or endorsed by the publisher.
