# Supplementary figures and images for: Enzymatic Hydrolysis of Broken Rice Protein: Antioxidant Activities by Chemical and Cellular Antioxidant Methods
Source: Front Nutr. 2021 Dec 9;8:788078. doi: 10.3389/fnut.2021.788078 (PMC8698253; doi:10.3389/fnut.2021.788078)

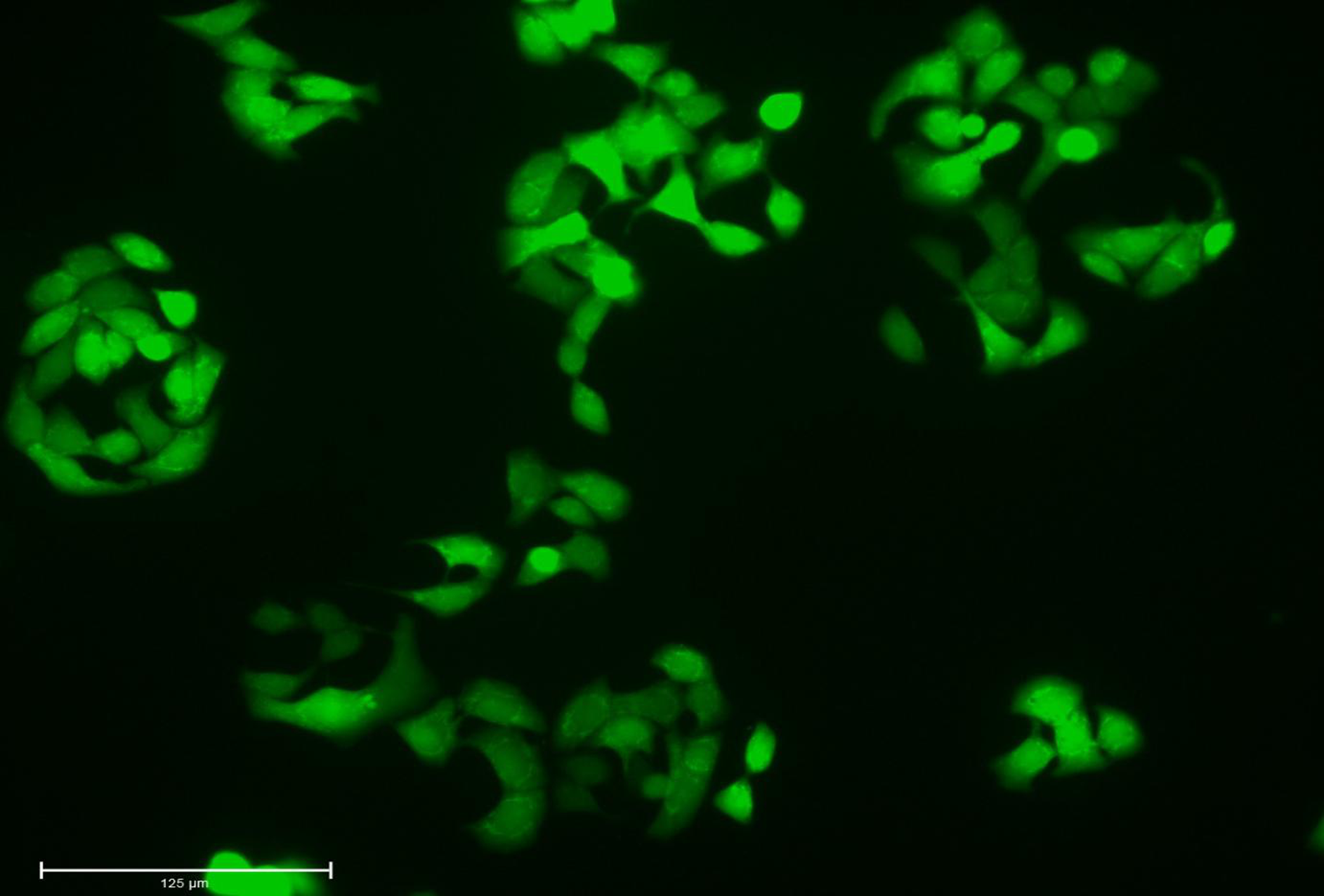

Supplement: Supplementary file 10 [file Image_1.TIF]

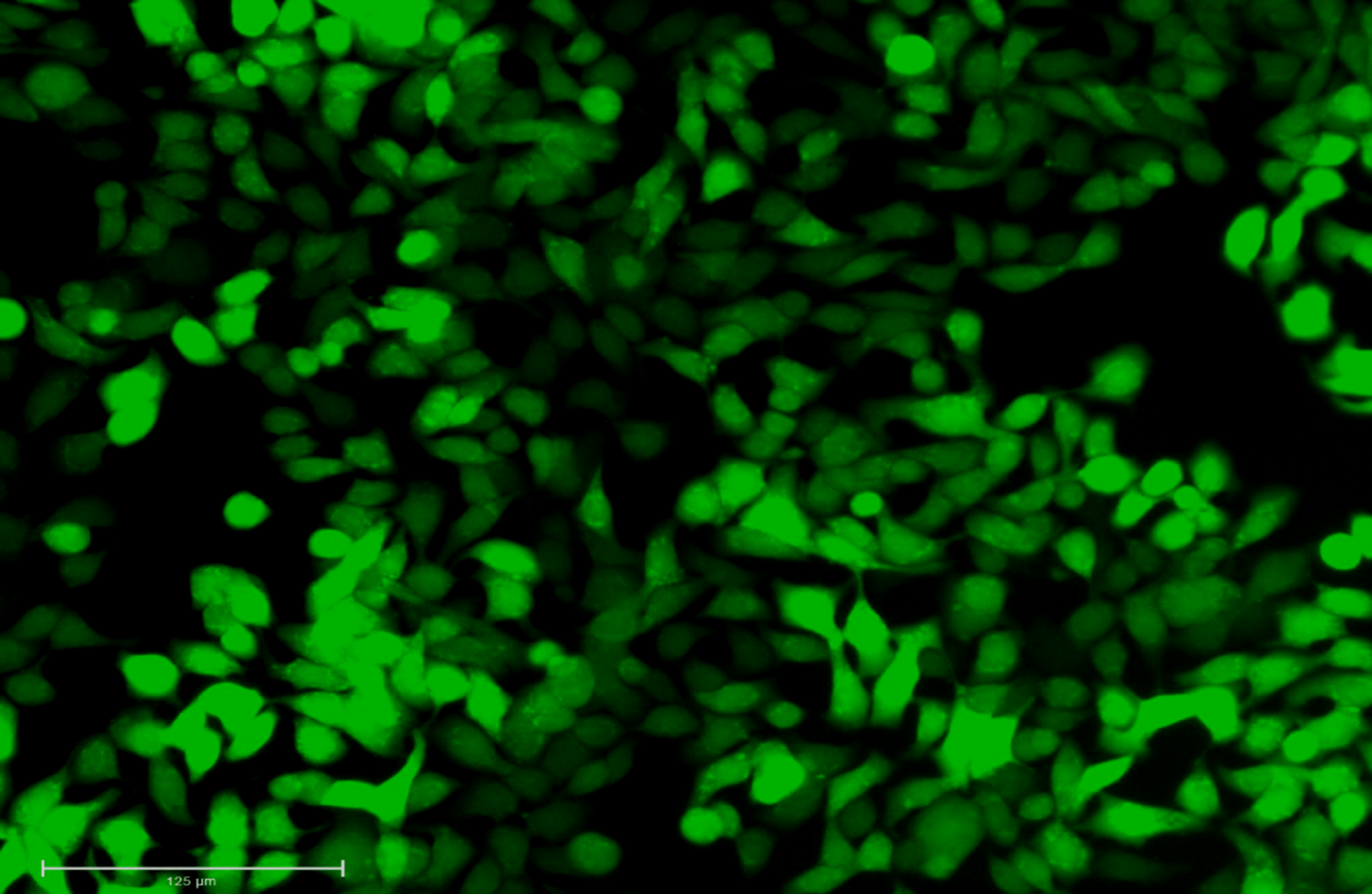

Supplement: Supplementary file 11 [file Image_2.TIF]

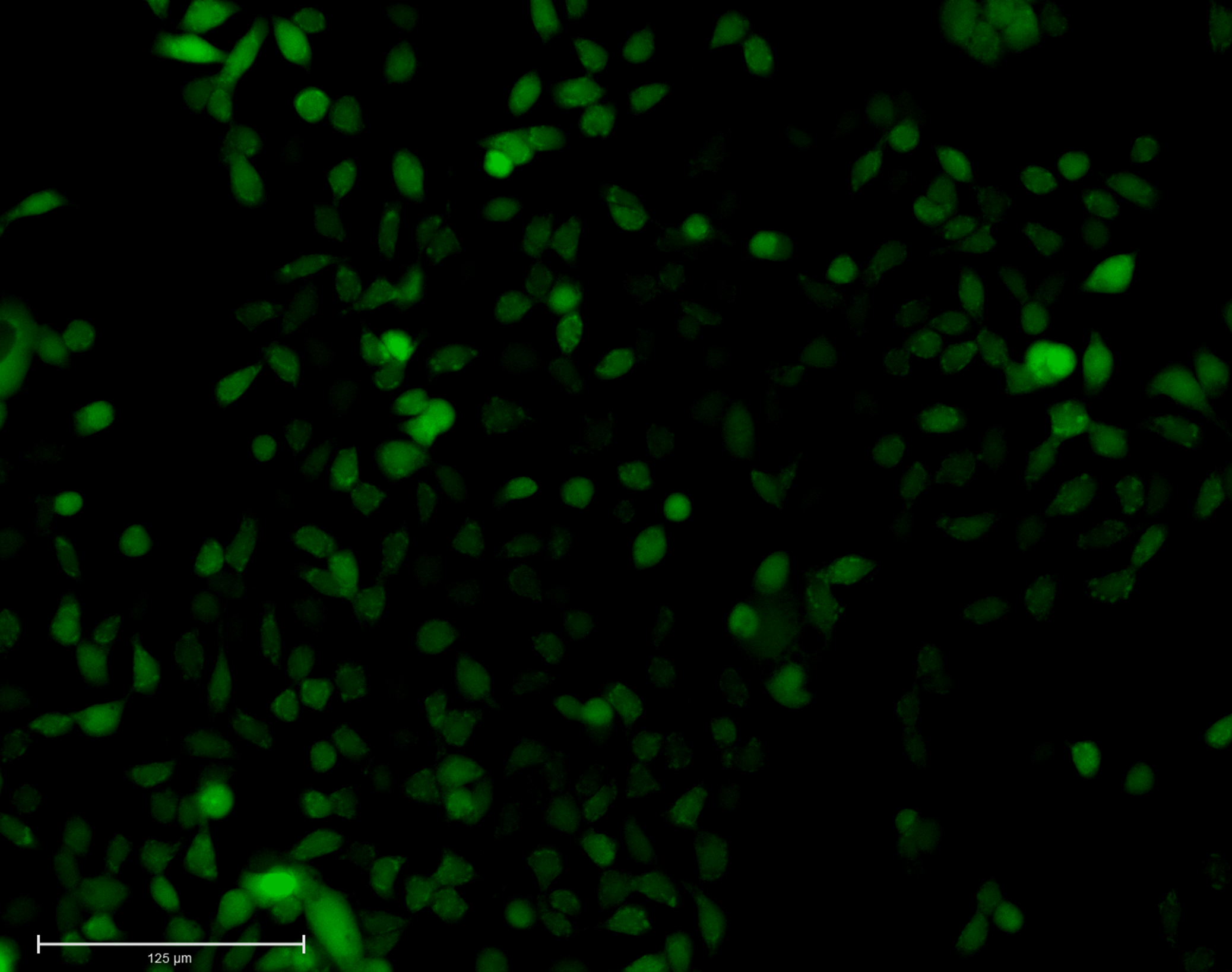

Supplement: Supplementary file 12 [file Image_3.TIF]

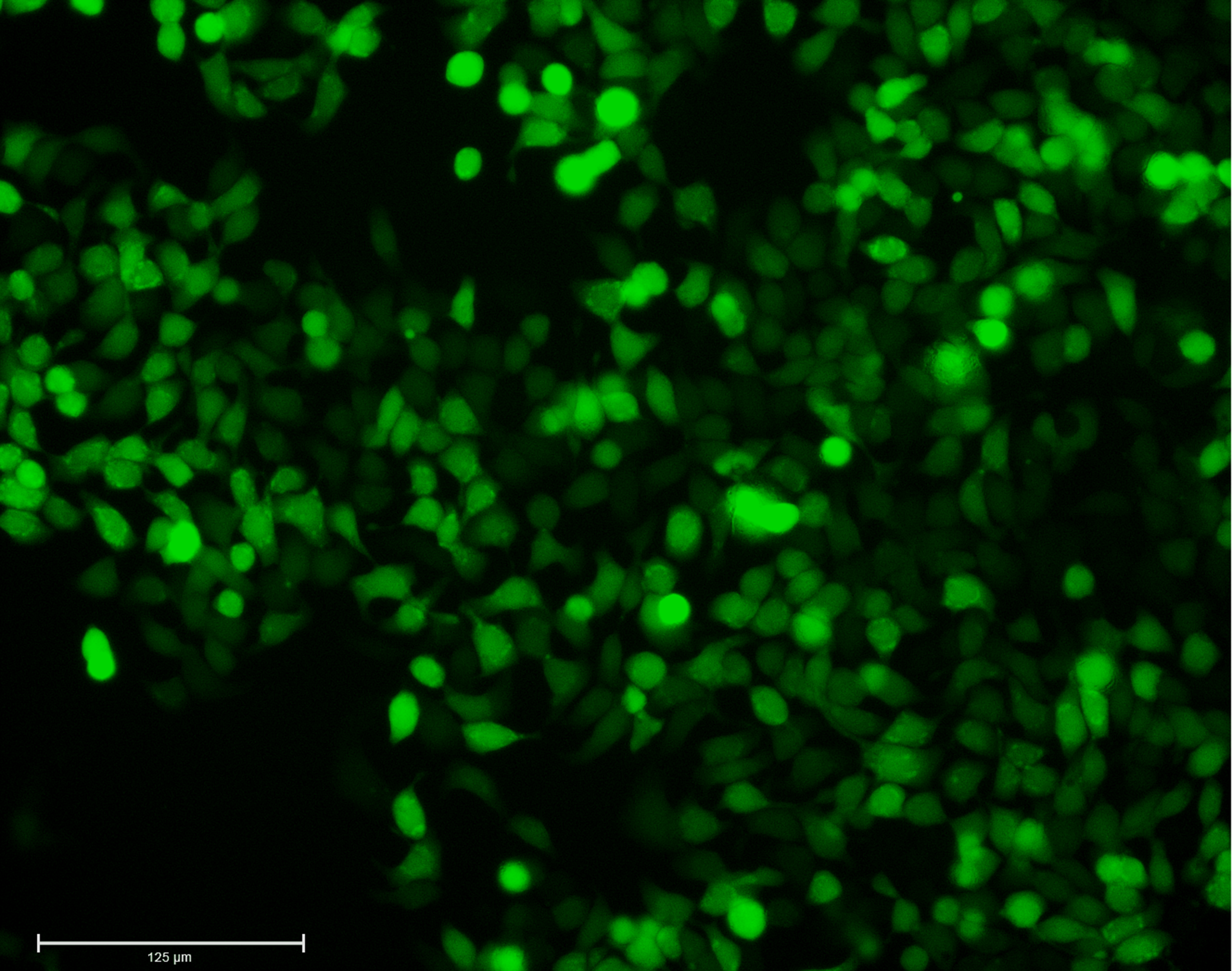

Supplement: Supplementary file 13 [file Image_4.TIF]

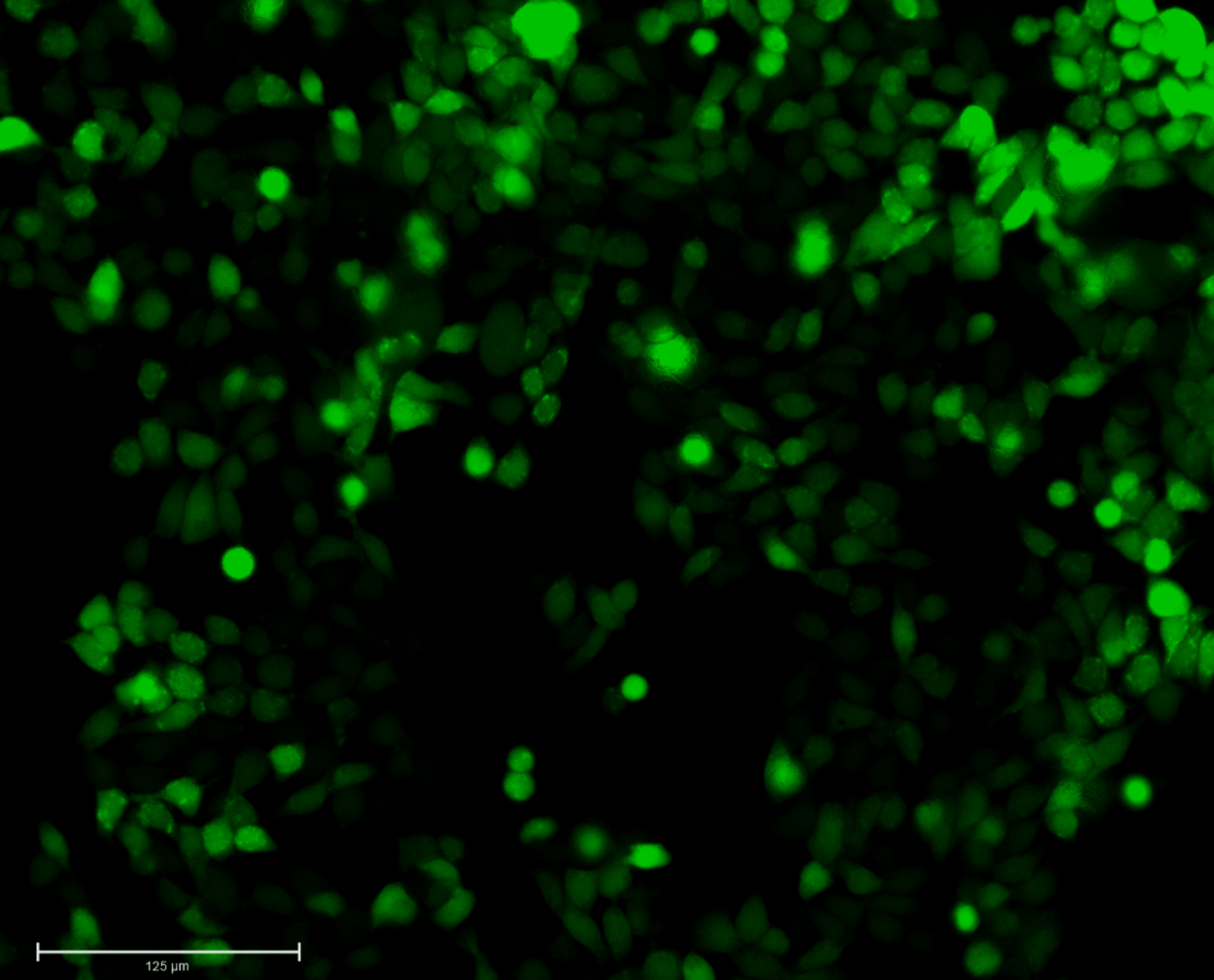

Supplement: Supplementary file 14 [file Image_5.TIF]

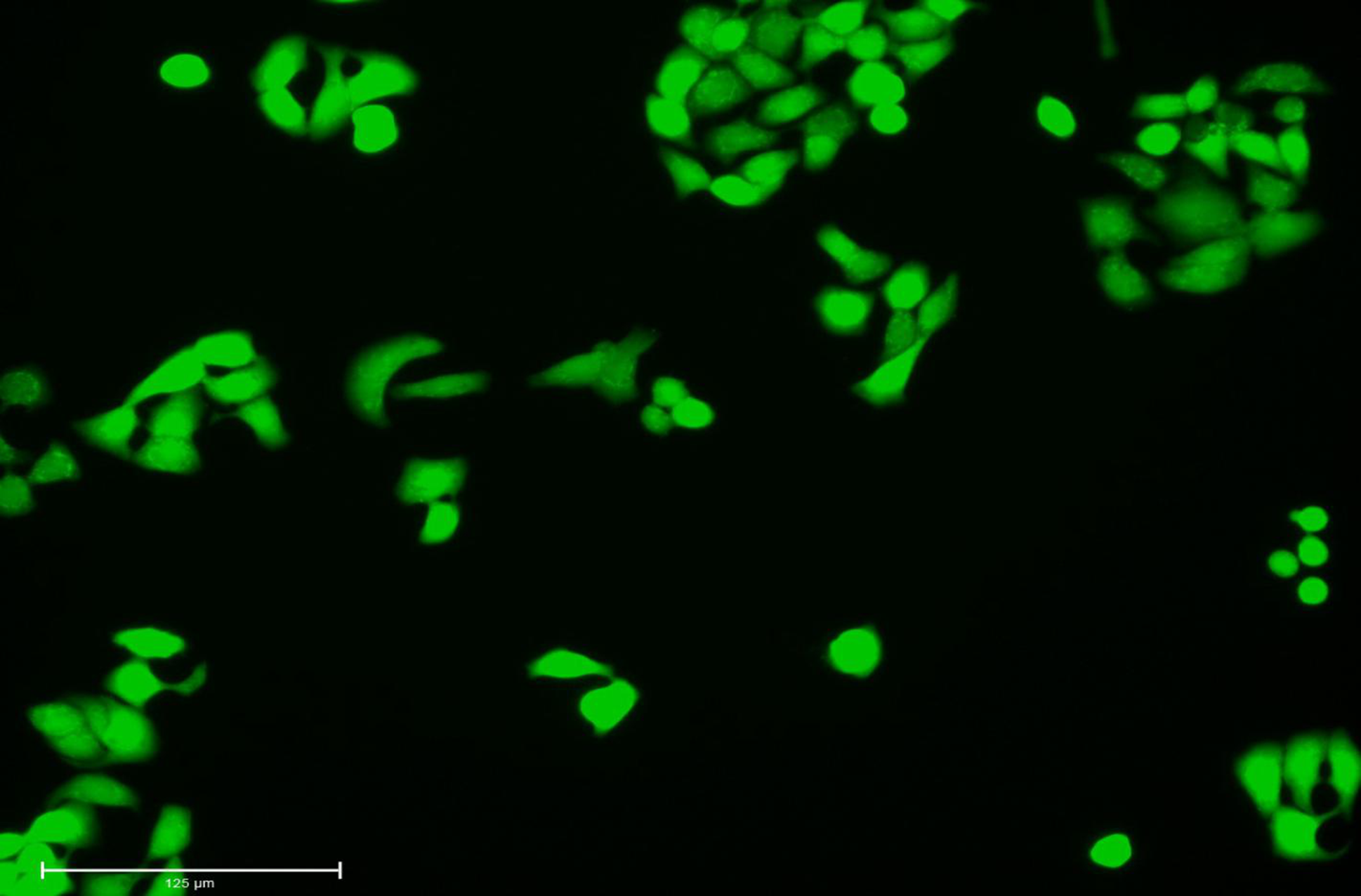

Supplement: Supplementary file 15 [file Image_6.TIF]

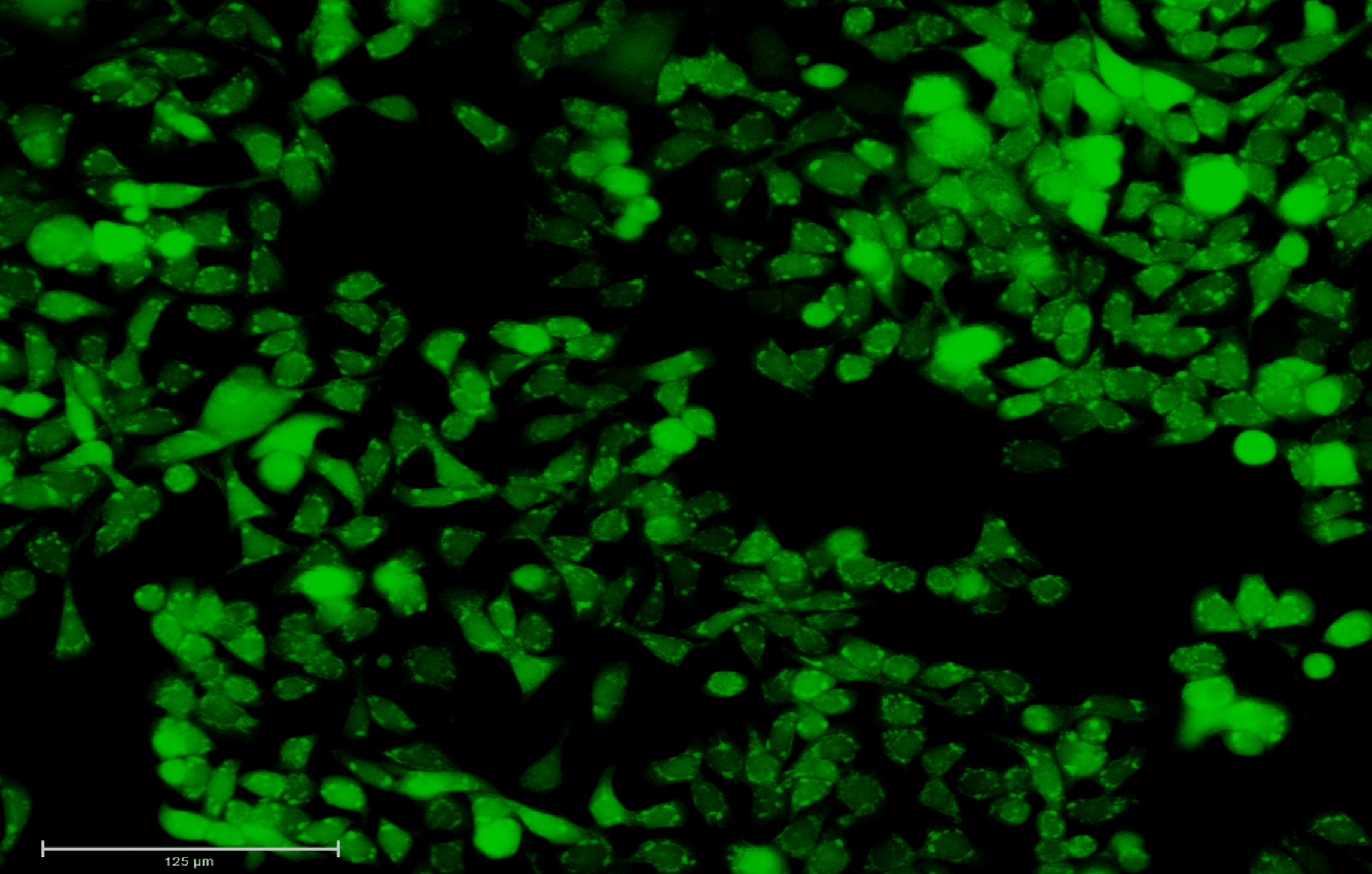

Supplement: Supplementary file 16 [file Image_7.TIF]

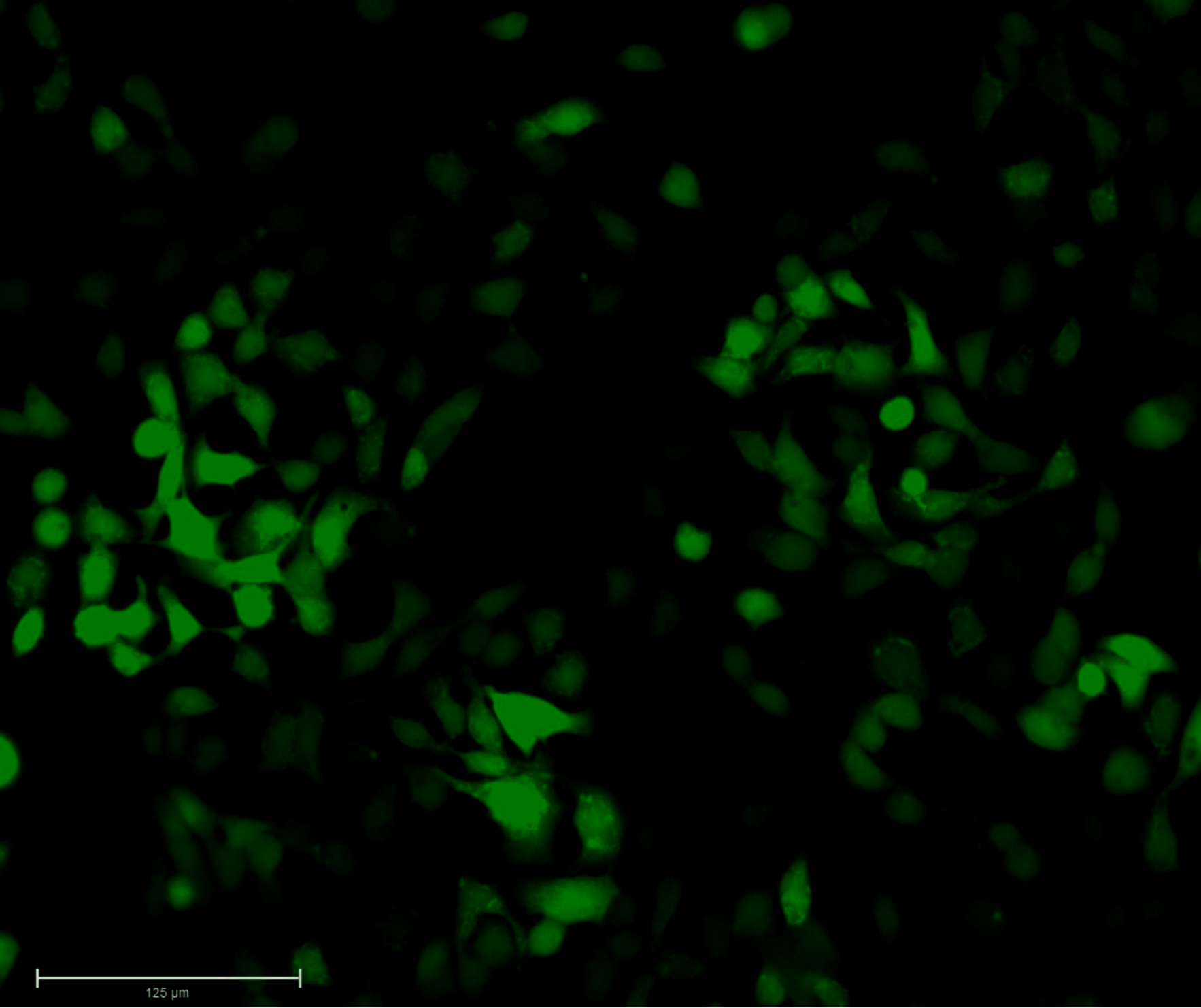

Supplement: Supplementary file 17 [file Image_8.TIF]

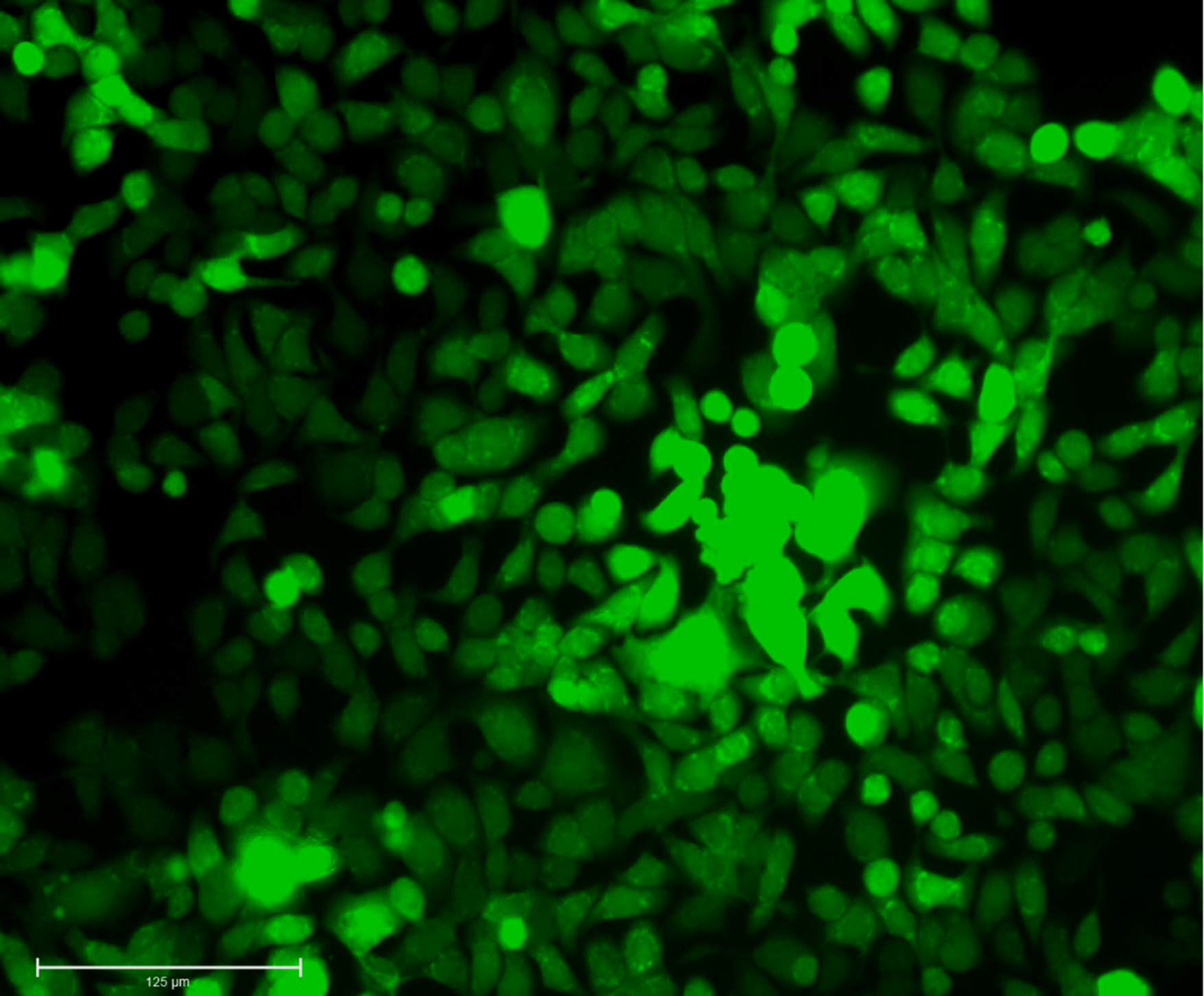

Supplement: Supplementary file 18 [file Image_9.TIF]

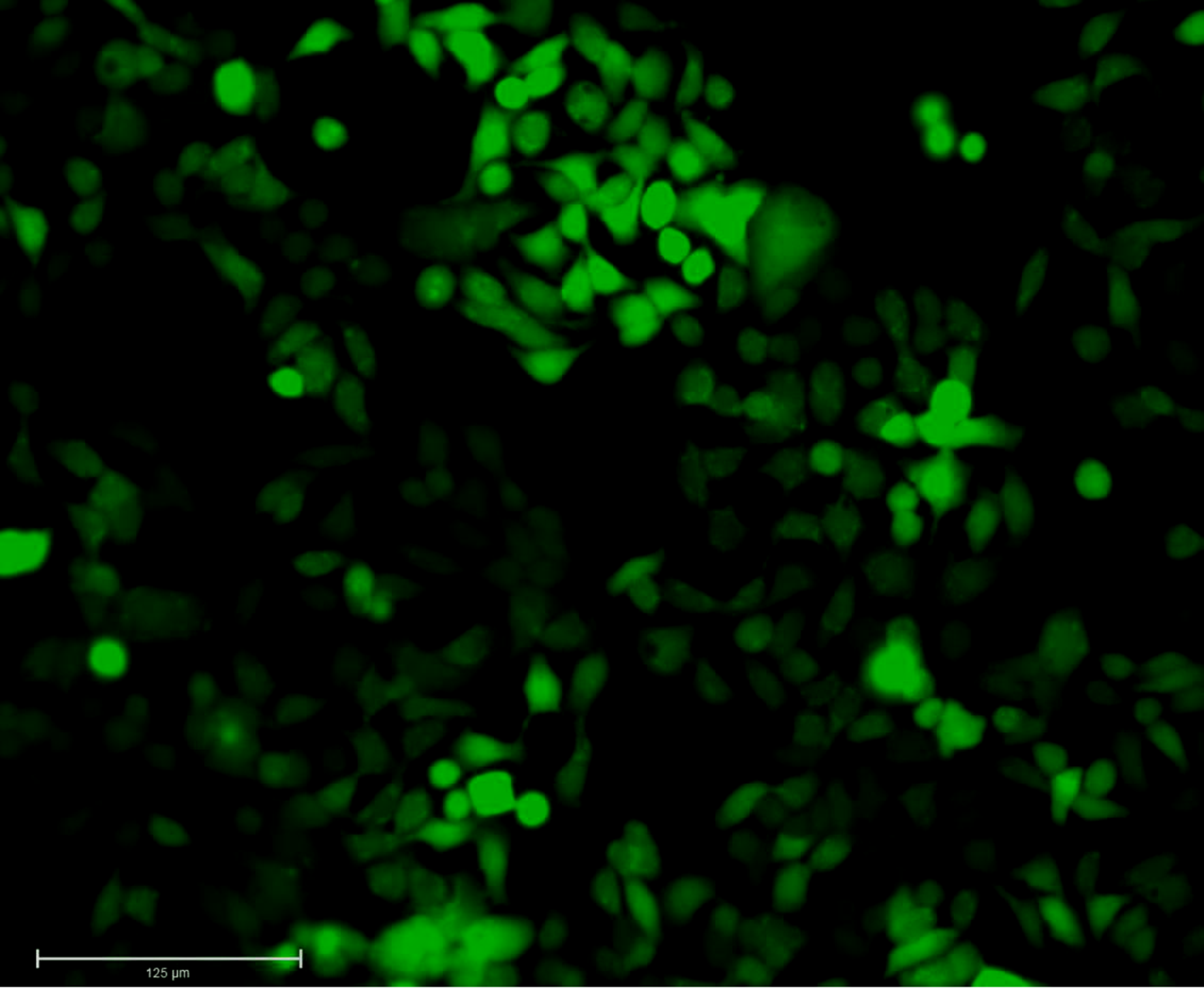

Supplement: Supplementary file 19 [file Image_10.TIF]

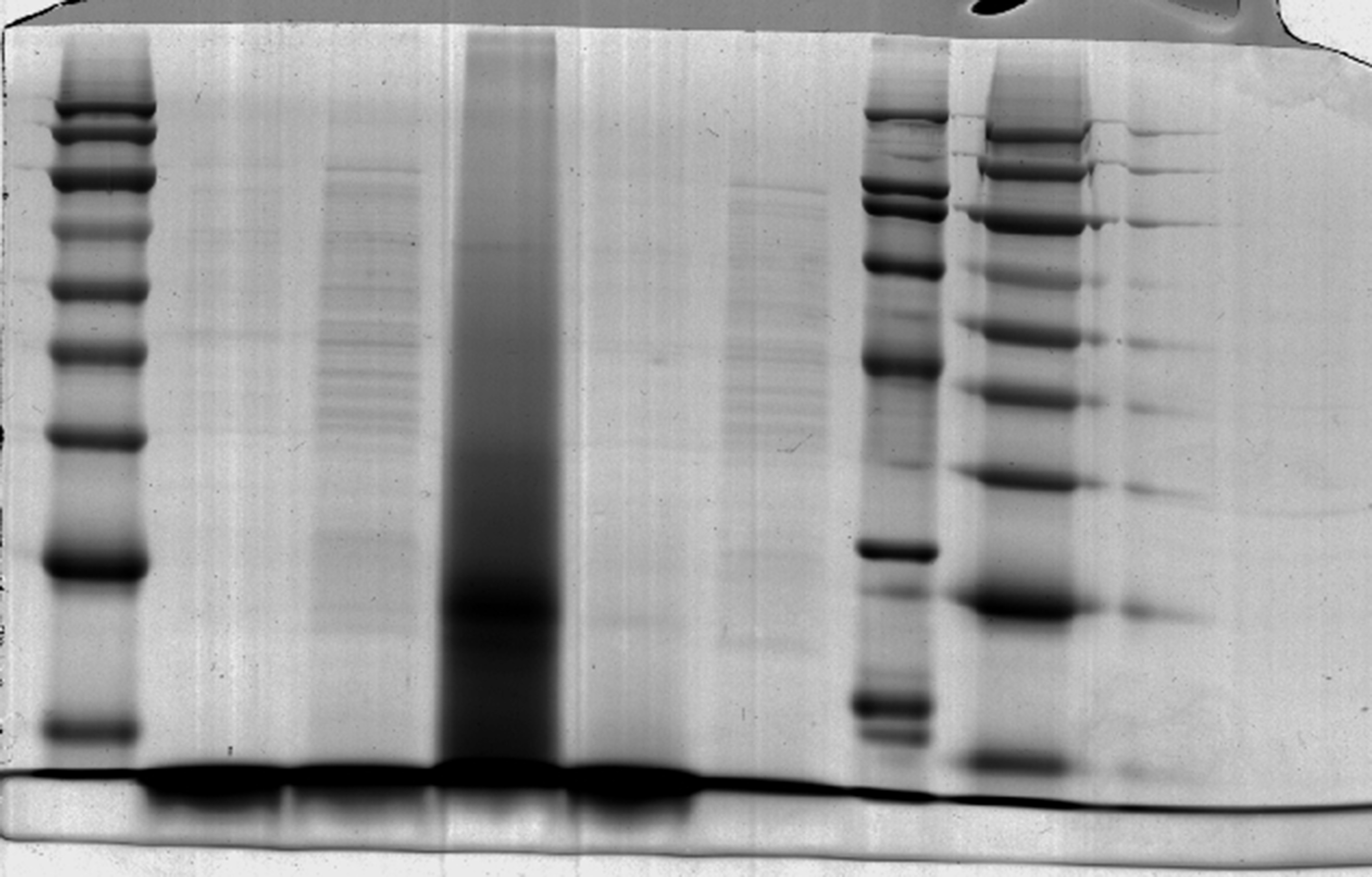

Supplement: Supplementary file 20 [file Image_11.JPEG]

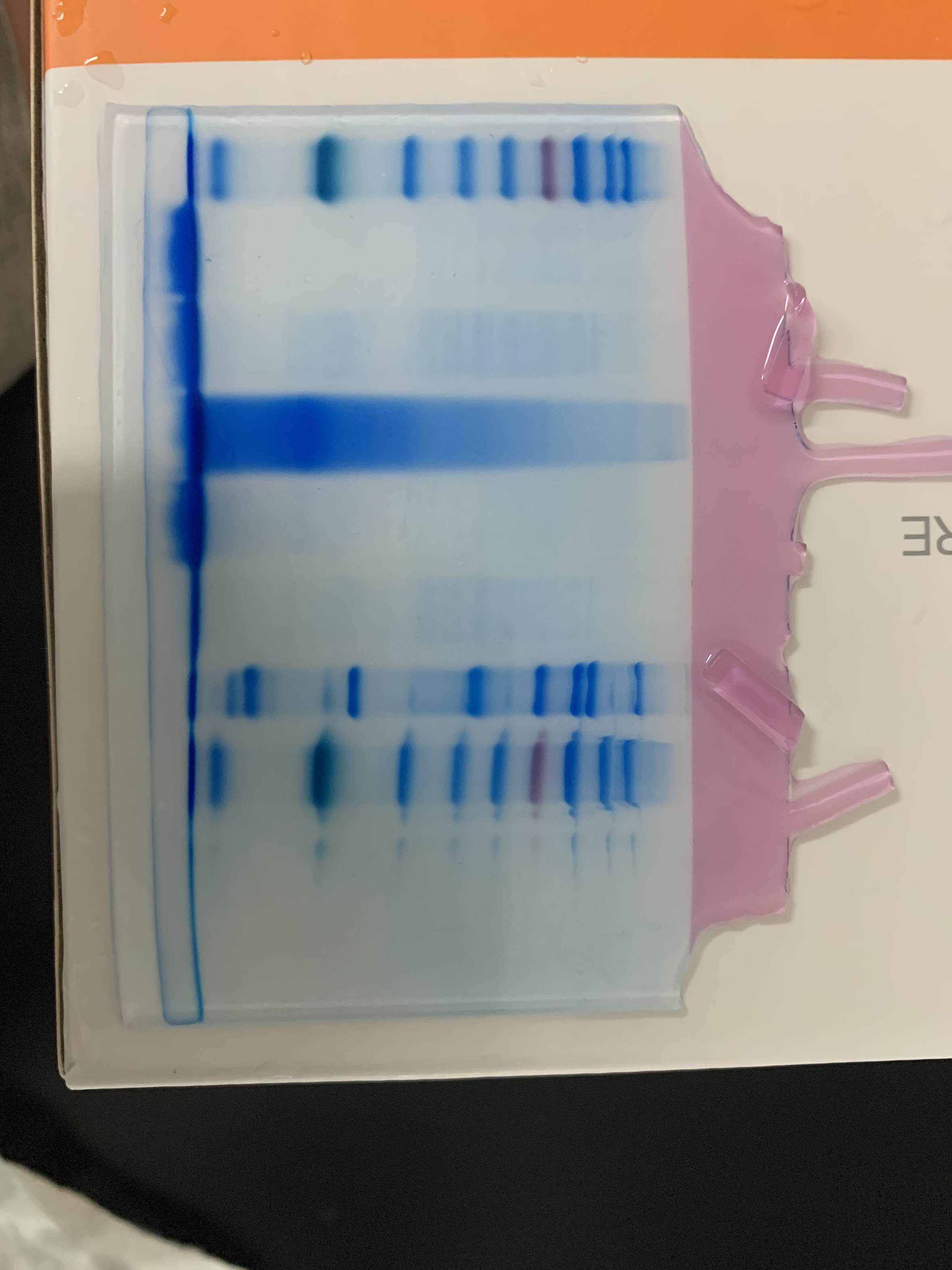

Supplement: Supplementary file 21 [file Image_12.JPEG]
